# Diverse Aquatic Animal Matrices Play a Key Role in Survival and Potential Virulence of Non-O1/O139 *Vibrio cholerae* Isolates

**DOI:** 10.3389/fmicb.2022.896767

**Published:** 2022-06-21

**Authors:** Lili Yan, Yinzhe Jin, Beiyu Zhang, Yingwei Xu, Xu Peng, Si Qin, Lanming Chen

**Affiliations:** ^1^Key Laboratory of Quality and Safety Risk Assessment for Aquatic Products on Storage and Preservation (Shanghai), Ministry of Agriculture and Rural Affairs of the People's Republic of China, Shanghai, China; ^2^College of Food Science and Technology, Shanghai Ocean University, Shanghai, China; ^3^Department of Biology, Archaea Centre, University of Copenhagen, Copenhagen, Denmark; ^4^Key Laboratory for Food Science and Biotechnology of Hunan Province, College of Food Science and Technology, Hunan Agricultural University, Changsha, China

**Keywords:** *V. cholerae*, aquatic product matrix, secretome, proteome, genome, virulence, resistance

## Abstract

*Vibrio cholerae* can cause pandemic cholera in humans. The waterborne bacterium is frequently isolated from aquatic products worldwide. However, current literature on the impact of aquatic product matrices on the survival and pathogenicity of *cholerae* is rare. In this study, the growth of eleven non-O1/0O139 *V. cholerae* isolates recovered from eight species of commonly consumed fish and shellfish was for the first time determined in the eight aquatic animal matrices, most of which highly increased the bacterial biomass when compared with routine trypsin soybean broth (TSB) medium. Secretomes of the *V. cholerae* isolates (draft genome size: 3,852,021–4,144,013 bp) were determined using two-dimensional gel electrophoresis (2DE-GE) and liquid chromatography-tandem mass spectrometry (LC-MS/MS) techniques. Comparative secretomic analyses revealed 74 differential extracellular proteins, including several virulence- and resistance-associated proteins secreted by the *V. cholerae* isolates when grown in the eight matrices. Meanwhile, a total of 8,119 intracellular proteins were identified, including 83 virulence- and 8 resistance-associated proteins, of which 61 virulence-associated proteins were absent from proteomes of these isolates when grown in the TSB medium. Additionally, comparative genomic and proteomic analyses also revealed several strain-specific proteins with unknown functions in the *V. cholerae* isolates. Taken, the results in this study demonstrate that distinct secretomes and proteomes induced by the aquatic animal matrices facilitate *V. cholerae* resistance in the edible aquatic animals and enhance the pathogenicity of the leading waterborne pathogen worldwide.

## Introduction

*Vibrio cholerae* is a Gram-negative bacterium that is found growing in brackish coastal waters and estuaries worldwide (Yen and Camilli, 2017). Ingestion of water or aquatic products contaminated with toxic *V. cholerae* can cause cholera in humans (Ramamurthy et al., 2020). In the Indian subcontinent, the description of a disease resembling cholera has been mentioned in Sushruta Samita, estimated to have been written between ~400 and 500 BC (Siddique and Cash, 2014). In 1817, the first pandemic of cholera spread from India to several other regions of the world (Lippi et al., 2016). Consequently, six additional major pandemics have occurred, the latest of which originated in Indonesia in the 1960s and was still ongoing (Rabaan, 2019). It was estimated that cholera caused ~95,000 deaths annually in endemic countries, such as India, Ethiopia, and Nigeria (Ali et al., 2015). To date, *V. cholerae* isolates have been classified into more than 200 serogroups (Zmeter et al., 2018), of which serogroups O1 and O139 can cause cholera outbreaks. Nevertheless, clinical infections with non-O1/O139 *V. cholerae* isolates have been reported (Zmeter et al., 2018). These pathogens can cause intestinal, extra-intestinal diseases, and even death (Zmeter et al., 2018). For example, a pathogenic and non-O1/O139 *V. cholerae* AM-19226 strain colonized human intestinal epithelial cells by a Type III secretory system (T3SS), disrupted homeostasis, and caused diarrhea disease (Miller et al., 2016). Virulence-related proteins have been identified in non-O1/O139 *V. cholerae* isolates, e.g., a cholix toxin (Chx), a multifunctional autoprocessing repeats-in-toxin (MARTX), a GlcNAc binding protein A (GbpA), a *V. cholerae* cytolysin (VCC), and an outer membrane protein OmpU (Ramamurthy et al., 2020). For instance, intraperitoneal injection of Chx in mice can cause a lethal hemorrhagic inflammatory and cytotoxic response in the liver (Ogura et al., 2017). Non-O1/O139 *V. cholerae* isolates exerted cell rounding *via* MARTX that induced the depolymerization of actin fibers of host cells (Fullner and Mekalanos, 2000). The binding of *V. cholerae* by GbpA led to increased mucus production, which drew more bacteria for better colonization of the host intestinal surface (Rothenbacher and Zhu, 2014). VCC was a membrane-damaging protein toxin with potent cytolytic/cytotoxic activity against a wide range of eukaryotic cells (Kathuria and Chattopadhyay, 2018), while OmpU was a key adhesion protein and an important virulence factor for the successful colonization of *Vibrio* species into the host (Ganie et al., 2021). Therefore, the identification of virulence determinants in non-O1/O139 *V. cholerae* isolates is imperative to ensure food safety systems and human health.

Several types of secretory systems have been identified in pathogenic bacteria (Ratner et al., 2017). Secreted proteins play very important roles in bacterial signaling, cell to cell communication, and survival in the environment and hosts (Stastna and Van Eyk, 2012). For instance, cholera toxin (CT), a major toxin of *V. cholerae*, is secreted by a Type II secretory system (T2SS) (Rasti and Brown, 2019). *V. cholerae* AM-19226 that lacks CT and toxin coregulated pilus (TCP), a type IV pilus required for *V. cholerae* pathogenesis, can cause enterotoxicity by T3SS pathogenic islands similar to that in pathogenic *Vibrio parahaemolyticus* (Megli and Taylor, 2013; Rivera-Cancel and Orth, 2017). Bacterial Type VI secretory system (T6SS) can also secret virulence factors (Miller, 2013), such as a hemolysin co-regulatory protein (Hcp) and a valine-glycine repeat protein G (VgrG) (Li et al., 2019). For example, three VgrG alleles have been reported in *V. cholerae*: VgrG-1 exhibited actin cross-linking activity with cytotoxic effect on eukaryotic cells; VgrG-2 was essential in regulating bacterial movement and biofilm formation; and VgrG-3 showed an antibacterial function by hydrolyzing cell wall of Gram-negative bacteria (Sha et al., 2013; Arteaga et al., 2020).

Two-dimensional gel electrophoresis (2D-GE) is one of the most versatile and widely used techniques to study the proteomics of a biological system (Meleady, 2018). The combination of 2D-GE with liquid chromatography-tandem mass spectrometry (LC-MS/MS) can provide basic information from protein identity to sample heterogeneity analysis. For instance, Mir et al. identified 150 differentially regulated proteins of virulent *Salmonella enterica* serovar Typhi in a model host of *Caenorhabditis elegans* using 2D-GE analysis (Mir et al., 2021). Monteiro et al. (2016) identified several differential cytoplasmic proteins between drug-resistant *Escherichia coli* 5A, 10A, 12A, and 23B strains and non-resistant *E. coli* L137 using 2D-GE combined with MS analysis, including a beta-lactamase Tem, an outer membrane protein A, and a beta-lactamase Toho-2. Zhu et al. (2020) obtained secretomes of 12 *V. parahaemolyticus* isolates and identified 8 virulence-associated proteins using 2D-GE and LC-MS/MS techniques, including a superoxide dismutase, a maltoporin, an outer membrane channel TolC, an enolase, an elongation factor Tu, a polar flagellin B/D, a transaldolase, and a flagellin C. Recently, Shan et al. (2021) determined secretomes and proteomes of 20 *V. cholerae* isolates by 2D-GE and LC-MS/MS analyses, and identified 11 extracellular and 22 intracellular virulence-associated proteins.

In our previous studies, 2D-GE conditions have been optimized recently (Zhu et al., 2020). Secretomes of *V. parahaemolyticus* isolates recovered from 12 species of edible aquatic animals were characterized, and differentially secreted proteins were identified using 2D-GE and LC-MS/MS techniques (Zhu et al., 2020). China is the largest producer, exporter, and consumer of aquatic products worldwide. The total aquatic product output was ~65,490,200 tons in China in 2020, which accounted for nearly 60% of the world's total output (National Bureau of Statistics, http://www.stats.gov.cn/, accessed on 20 August 2021). Recently, several *V. cholerae* isolates were recovered from 36 species of edible aquatic animals and identified by our research group (Chen et al., 2021). Secretomes and proteomes of 20 *V. cholerae* isolates incubated in the routine trypsin soybean broth (TSB) medium were determined using 2D-GE and LC-MS/MS techniques (Shan et al., 2021). Based on our previous studies, we, therefore, asked whether various aquatic product matrices could affect the survival and pathogenicity of *V. cholerae* isolates of aquatic animal origins. Thus, the major objectives of this study were: (1) to determine the growth of eleven non-O1/O139 *V. cholerae* isolates in eight types of commonly consumed fish and shellfish matrix media; (2) to employ 2D-GE and LC-MS/MS techniques to obtain secretomes and proteomes of these *V. cholerae* isolates when incubated in the matrix media; and (3) to identify virulence- and resistance-associated proteins of the *V. cholerae* isolates induced by the matrices. To the best of our knowledge, this study was the first to investigate the impact of various aquatic animal matrices on the survival, secretomes, and proteomes of *V. cholerae* isolates. The results of this study will support the increased demand for new vaccine targets for food safety control of *V. cholerae* contamination in edible aquatic animals.

## Materials and Methods

### *V. cholerae* Isolates and Culture Conditions

The non-O1/O139 *V. cholerae* strains used in this study (Table 1) were isolated and characterized by Su and Chen (2020), and stored at −80°C freezer in our laboratory at Shanghai Ocean University, Shanghai, China. *V. cholerae* isolates (Table 1) were inoculated in TSB (Beijing Luqiao Technology Co., Ltd., Beijing, China) (pH 8.5, 3% of NaCl) or aquatic product matrix media (see below), and incubated at 37°C, respectively. Growth curves of *V. cholerae* isolates were determined using Bioscreen Automatic Growth Curve Analyzer (BioTek Instruments, Inc., Winooski, VT, USA). Bacterial cells grown to the mid-logarithmic phase without shaking were collected by centrifugation for extracellular protein extraction, or to the late logarithmic phase shaking at 180 rpm for intracellular protein extraction as described previously (Zhu et al., 2020).

**Table 1:** The *V. cholerae* isolates and aquatic product matrices used in this study.

***V. cholerae* isolate**	**Aquatic product matrix**		
	**Species of aquatic animal**	**Common name**	**Type of aquatic product**
b9-50	*Mactra antiquata*	Triangular clam	Shellfish
B1-31	*Parabramis pekinensis*	Bream	Fish
B8-16	*Parabramis pekinensis*	Bream	Fish
J9-62	*Carassius auratus*	Crucian carp	Fish
L10-6	*Aristichthys nobilis*	Silver carp	Fish
N3-6	*Paphia undulata*	Corrugated buffy clam	Shellfish
N4-21	*Perna viridis*	Mussel	Shellfish
N8-56	*Mactra quadrangularis Deshayes*	Clam	Shellfish
N8-88	*Mactra quadrangularis Deshayes*	Clam	Shellfish
Q6-10	*Ctenopharyngodon idellus*	Grass carp	Fish
Q10-54	*Ctenopharyngodon idellus*	Grass carp	Fish

### Preparation of Aquatic Product Matrix Media

The eight species of edible aquatic animals included 4 species of fish: *Aristichthys nobilis, Carassius auratus, Ctenopharyngodon idellus*, and *Parabramis pekinensis*; and 4 species of shellfish: *Mactra antiquata, Mactra quadrangularis Deshayes, Perna viridis*, and *Paphia undulata* (Table 1). The fresh aquatic animals were sampled from Shanghai Luchao Port Aquatic Market (30°51′40.21″ N, 121°50′48.40″ E) in Shanghai, China in March and May of 2021. The samples were collected in sterile sealed bags (Nanjing Maojie Microbial Technology Co., Ltd., Nanjing, China), and immediately packed in iceboxes (700 × 440 × 390 mm) and transferred to the laboratory at Shanghai Ocean University for analysis.

Aquatic product matrix media were prepared according to the method described by Wang et al. (2017) with minor modifications. Briefly, aliquots of 200 g (wet weight) of fish meat samples (fish skin was removed) were cut into 2 × 2 × 2 cm pieces using sterile knives, while ~100 g (wet weight) of shellfish meat samples (shell was removed) were washed with sterile water. The processed samples were collected in sterile sealed plastic bags (Maojie, Nanjing, China), and stored at −20°C for 4 days. Subsequently, the samples were transferred to 4°C for 3 days to collect the matrix leach solution. After freezing and thawing twice, the collected matrix leach solution was centrifuged at 10,000 g for 20 min at 4°C, and the supernatant was filtered through a.22-μm-pore membrane filter (Millipore, Bedford, MA, USA). The sterile filtrate derived from each of the eight types of aquatic animal samples was stored at −80°C and used as matrix media (Table 1). The matrix leaching rate was calculated by the percentage of leaching solution mass to the processed sample mass. All tests were performed in triplicates.

### Measurement of Crude Protein, Carbohydrate, and Fat Components

Protein concentrations of the matrix media were measured using Bradford Protein Assay Kit (Shanghai Sangon Biological Engineering Technology and Service Co., Ltd., Shanghai, China) according to the manufacturer's instructions, and serum albumin was used as the standard protein. Crude fat contents were determined using Automatic Soxhlet Fat Extraction System (Gerhardt, Bonn, Germany) (Shin et al., 2013). Carbohydrate contents were measured by the phenol–sulfuric acid method using Synergy 2 Multifunctional Microplate Reader (BioTek, Maricopa, USA) (Albalasmeh et al., 2013). The pH was measured using an electronic pH meter (Mettler Toledo FiveEasy Plus, Shanghai, China). All tests were performed in triplicates.

### 2D-GE Analysis

Extracellular proteins of the *V. cholerae* isolates were extracted according to the method described previously (Zhu et al., 2020). Intracellular proteins were extracted using Bacterial Protein Extraction Kit (Sangon, Shanghai, China) containing protease inhibitors, following the manufacturer's instructions.

The 2D-GE analysis was performed according to the method described previously (Zhu et al., 2020) with minor modifications: a 200 μL of pyrolysis buffer, including 8-M urea (Sangon, Shanghai, China), 4% (wt/vol) 3-[3-cholamidopropylldimethylammono-1-prosulfonate] (CHAPS, Sangon, Shanghai, China), 65-mM disulfide threitol (DTT, Sangon, Shanghai, China), and 2% (vol/vol) of Bio-Lyte 3/10 ampholyte (Bio-Rad Laboratories Inc, Hercules, CA, USA), was added into 20 μL/μg of extracellular protein sample. The undissolved residues were removed by centrifugation at 12,000 g for 15 min at 4°C. Isoelectric focusing (IEF) was performed using immobilized pH gradient (IPG) gels (pH 4–7, 7 cm; Bio-Rad, Hercules, USA), on which a 150 μL of the sample was loaded.

After hydration, IEF and SDS-PAGE analyses, the gels were stained using Protein Stains K (Sangon, Shanghai, China) according to the manufacturer's instructions. Silver-stained gels were scanned, and protein spot detection, matching, and quantitative intensity analysis were performed using PDQuest Advanced 8.0.1 software (Bio-RAD, Hercules, USA), as described previously (Zhu et al., 2020; Shan et al., 2021).

### LC-MS/MS Analysis

The LC-MS/MS analysis was carried out at HooGen Biotech, Shanghai, China using Q Executive Mass Spectrometer (Thermo Fisher Scientific (TFS), Waltham, MA, USA) coupled with Easy nLC 1200 Chromatography System with the same parameters described previously (Zhu et al., 2020). The automated peptide identification using UniProt *V. cholerae* 89344 20210707 databases (download in July 2021) in Mascot version 2.2 server (Matrix Science, London, United Kingdom), as described previously (Zhu et al., 2020; Shan et al., 2021).

### Comparative Genome Analysis

Draft genome sequences of the eleven *V. cholerae* isolates were determined in our previous research (Shan et al., 2021). Approximately 49,073 to 121,091 clean single reads were obtained, and the genome sequencing depth was 248.74-fold to 331.34-fold coverage (Shan et al., 2021). Comparative genomic analyses were carried out in this study. Virulence- and resistance-related genes, as well as transporter genes, were predicted using Diamond software (https://www.crystalimpact.de/diamond) against Virulence Factor Database of Bacterial (VFDB) (http://www.mgc.ac.cn/VFs/), Comprehensive Antibiotic Resistance Database (CARD) (http://arpcard.Mcmaster.ca), and Transporter Classification Database (TCDB) (http://www.tcdb.org/), respectively. Gene coding for proteins with signal peptides were predicted using signalP software (http://www.cbs.dtu.dk/services/SignalP), and transmembrane proteins were predicted using Tmhmm software (http://www.cbs.dtu.dk/services/TMHMM/). Prophage and CRISPR-Cas gene sequences were predicted using Phigaro (http://phage-finder.sourceforge.net/), and Minced (https://sourceforge.net/projects/minced/) software, respectively.

Genome circular maps of the eleven *V. cholerae* isolates were constructed based on the reference genome of *V. cholerae* MS6 (GenBank accession number: NZ_AP014524.1), which was retrieved from the Genome Database of National Center for Biotechnology Information (NCBI) (http://www.ncbi.nlm.nih.gov/genome). The Blastcluster software (http://www.ncbi.nlm.nih.gov/) was used for the pangenome analysis. Common and strain-specific genes were predicted using OrthoMCL software (http://orthomcl.org/common/downloads/software/).

### Quantitative Reverse Transcription-Polymerase Chain Reaction Assay

The qRT-PCR assay was performed according to the method described previously (Zhu et al., 2020). The 16S RNA was used as the internal reference gene (Zhu et al., 2020). All RT-PCRs were performed using CFX96 Real-Time PCR Detection System (Bio-Rad, Hercules, USA). In this study, all tests were performed in triplicate. The data were analyzed using SPSS statistical analysis software version 17.0 (SPSS Inc., Armonk, NY, USA).

## Results

### Genome Features of the Eleven *V. cholerae* Isolates Originating in Edible Aquatic Animals

**Figure 1:**
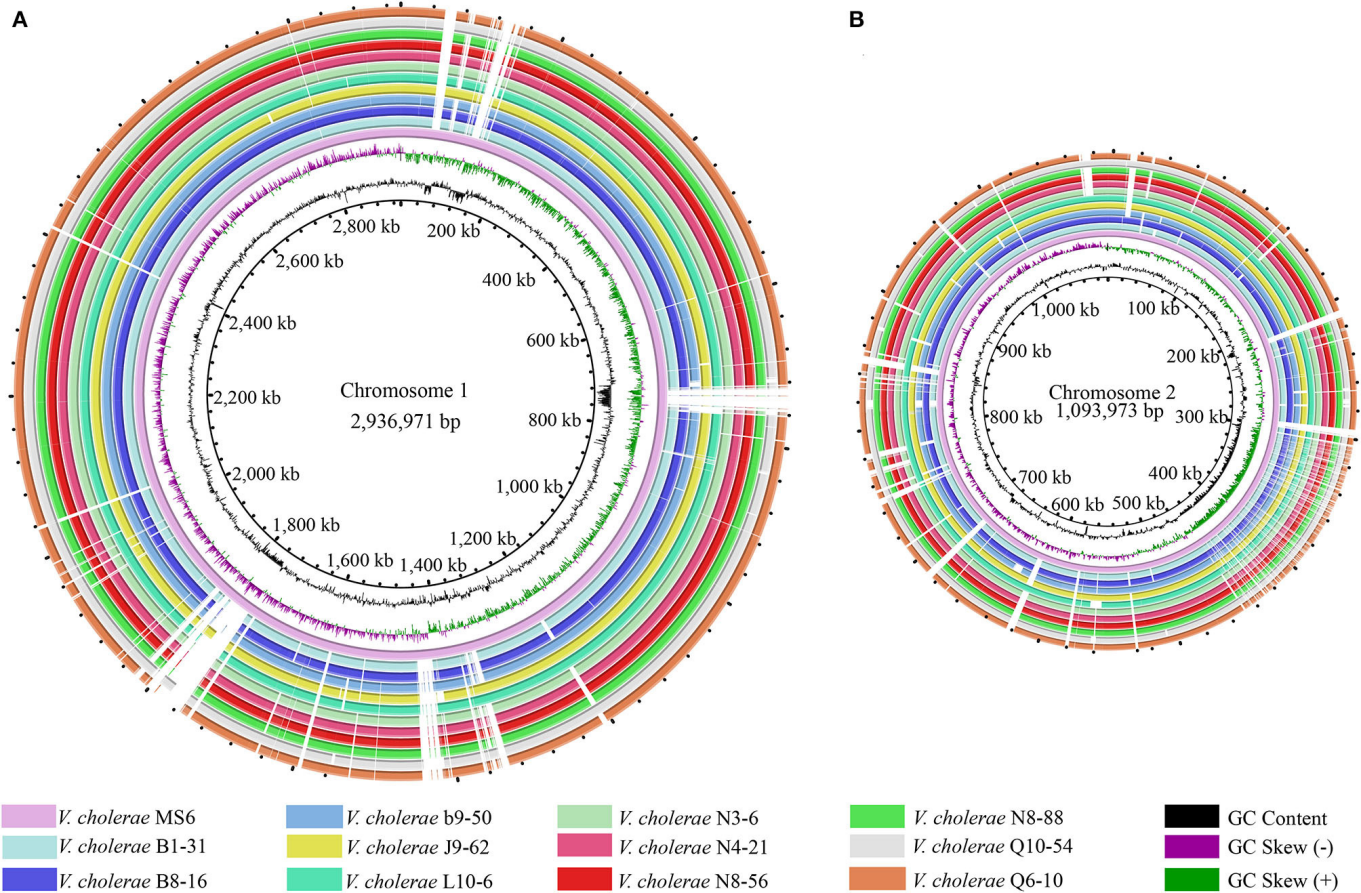
Genome circular maps of the eleven *V. cholerae* isolates. **(A,B)** The larger and smaller chromosomes of the *V. cholerae* genomes, respectively. *V. cholerae* MS6 was used as a reference genome (GenBank accession number: NZ_AP014524.1). Circles from the inward to outside represented GC-skew (values more than zero in purple and less than zero in green), GC content, predicted protein-coding genes of the reference genome, and eleven *V. cholerae* genomes, respectively.

Phenotypes of the eleven *V. cholerae* isolates were characterized (Su and Chen, 2020), which were recovered from eight species of commonly consumed fish and shellfish (Table 1). Draft genome sequences of these isolates ranged from 3,852,021 to 4,144,013 bp, with G+C contents from 47.37 to 47.7% (Shan et al., 2021). Remarkably, among the annotated 3,394–3,717 genes, ~524–559 were predicted as virulence-related genes against the VFDB in the eleven *V. cholerae* genomes, which referred to adhesion, invasion, damage of host cells and tissues, regulation of virulence, motility, biofilm formation, intracellular and extracellular survival, or nutrient acquisition (Webb and Kahler, 2008). Moreover, ~186–207 genes were predicted as resistance-related genes; ~322–347 genes coded for proteins with signal peptides; and 829–885 genes for transmembrane transporters in the eleven *V. cholerae* genomes. Additionally, several CRISPR-Cas repeat arrays (*n* = 2–6), and prophage gene clusters (*n* = 0–3), ranging from 9,415 to 33,111 bp, were identified (Supplementary Table S1), implying possible horizontal gene transfer during the genome evolution of these *V. cholerae* isolates. The genome circular maps of the eleven *V. cholerae* isolates were constructed (Figure 1).

Comparative genomic analyses revealed that *V. cholerae* b9-50 isolate, originating from the shellfish *M. antiquata*, had the largest genome size (4,144,013 bp), whereas *V. cholerae* L10-6 isolate, from the fish *A. nobilis*, was the smallest (3,852,021 bp). The maximum number of virulence-related genes (*n* = 559) were identified in *V. cholerae* J9-62 isolate from the fish *C. auratus*, whereas *V. cholerae* Q6-10 isolate from the fish *C. idellus* had the minimum (*n* = 524). Interestingly, *V. cholerae* J9-62 also carried the maximum number of resistance-related genes (*n* = 207), while *V. cholerae* N3-6, N4-21, N8-88, and Q6-10 isolates, which originated from the shellfish *P. undulata, P. viridis, M. quadrangularis Deshayes*, and fish *C. idellus*, respectively, contained the minimum (*n* = 186). *V. cholerae* b9-50 genome carried 3 prophage gene clusters (total length of 61,553 bp), whereas none was identified in *V. cholerae* L10-6, and Q10-54 from the fish *C. idellus*.

**Figure 2:**
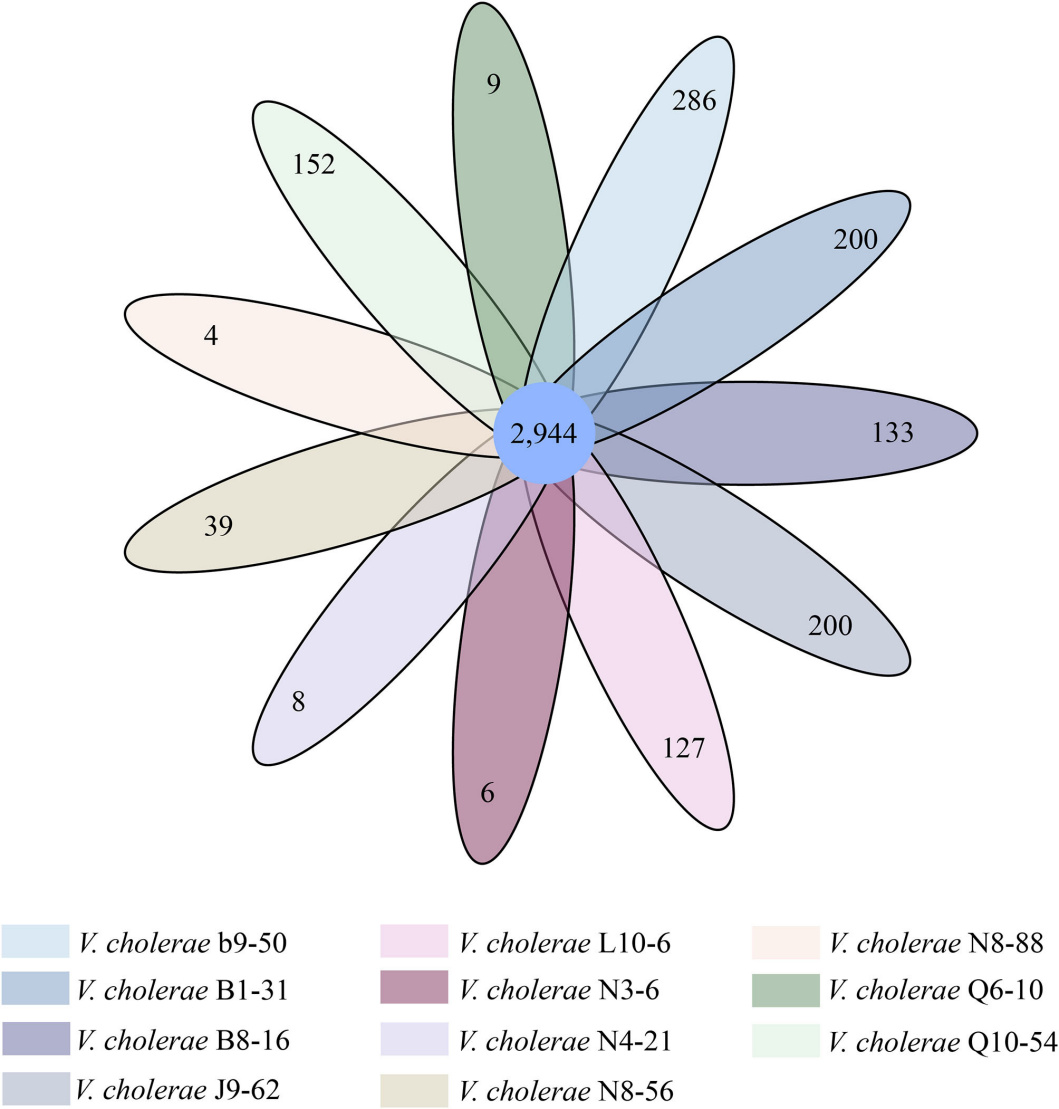
Venn diagram of the identified core and strain-specific genes of the 11 *V. cholerae* genomes.

Comparative genomic analyses also revealed 2,944 core genes shared by the eleven *V. cholerae* isolates. Approximately 4–286 strain-specific genes were identified (Figure 2). Notably, the highest number of strain-specific genes were identified in *V. cholerae* b9-50 (*n* = 286), whereas *V. cholerae* N8-88 contained the fewest (*n* = 4). Although a large number of the strain-specific genes in *V. cholerae* b9-50, B1-31, J9-62, L10-6, B8-16, Q10-54, and N8-56 genomes were predicted as unknown function in current databases, some of the functional genes were involved in bacterial virulence and resistance. For example, among the 286 strain-specific genes identified in *V. cholerae* b9-50 genome, approximately 57 of which matched the Gene Ontology (GO) database. Of these, 16 and 4 genes coded for virulence- and resistance-related proteins, respectively. Similarly, among the 200 strain-specific genes identified in *V. cholerae* B1-31 isolate originating from the fish *P. pekinensis*, ~44 of which matched the GO database. There were 9 and 3 genes coded for virulence- and resistance-related proteins, respectively. Conversely, only a few strain-specific genes were identified in *V. cholerae* N8-88, N3-6, N4-21, and Q6-10 genomes (*n* = 4–8).

### Preparation of Eight Types of Aquatic Product Matrix Media

**Figure 3:**
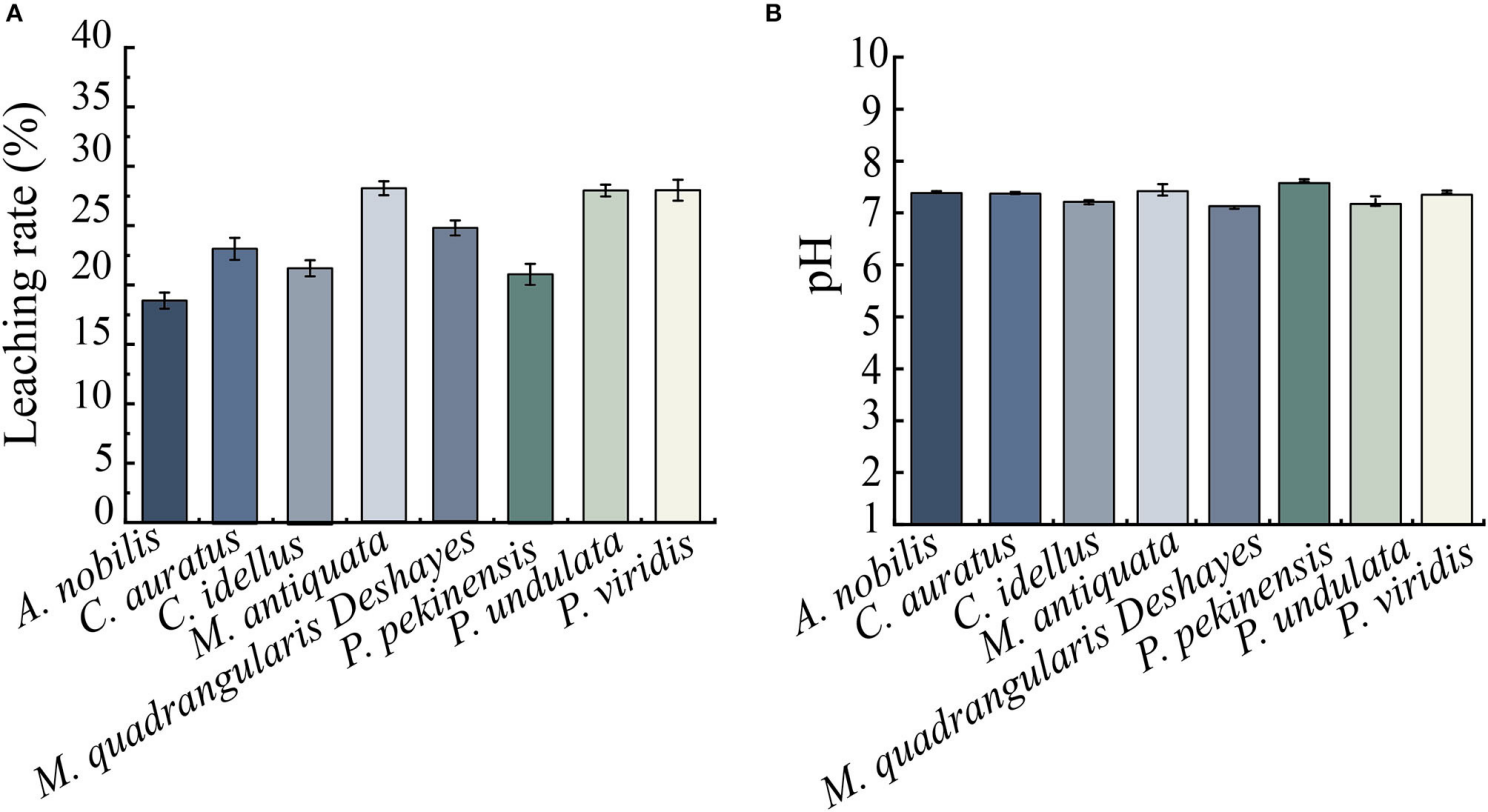
Leaching rate and initial pH values of the 8 types of aquatic product matrices. **(A)** Leaching rate. **(B)** Initial pH. Values were means ± S.D. of three parallel measurements.

Leaching rates and initial pH values of 8 types of aquatic product matrix media were determined, and the results are shown in Figures 3A,B. After repeated the freezing and thawing of aquatic products twice at −20°C for 4 days and 4°C for 3 days, leaching rates of the 8-matrix media ranged from 18.68 to 28.06%, among which the highest one was observed from the shellfish *M. antiquata* matrix (28.06%), followed by the shellfish *P. viridis* (28.01%), and *P. undulata* (27.97%) matrices. Conversely, the leaching rate of the fish *A. nobilis* matrix was the lowest (18.68%), followed by the fish *P. pekinensis* (20.97%) and *C. idellus* (21.57%) matrices.

As shown in Figure 3B, pH values of the 8-matrix media ranged from 7.09 to 7.60, among which the highest pH was observed from *P. pekinensis* matrix (pH 7.60), whereas the pH of *M. quadrangularis Deshayes* matrix was the lowest (pH 7.09).

### Key Components of Diverse Aquatic Product Matrix Media

**Figure 4:**
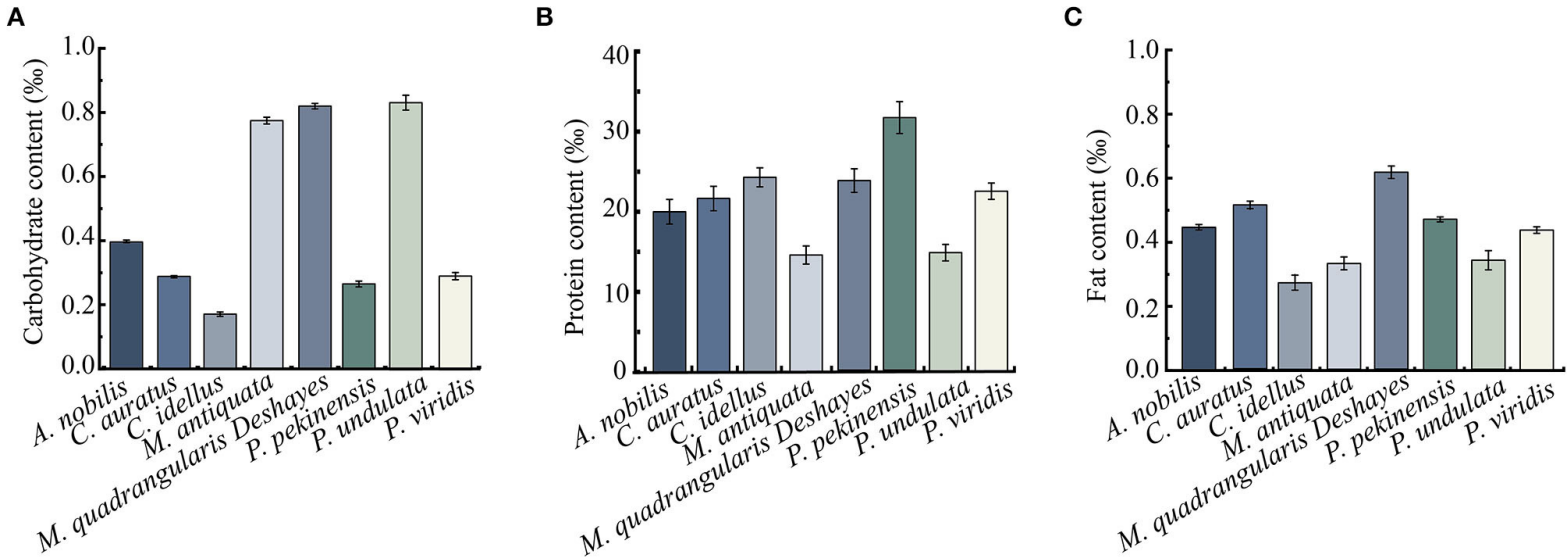
Carbohydrate, protein, and fat contents of the 8 types of aquatic product matrices. **(A)** Carbohydrate content. **(B)** Protein content. **(C)** Fat content. Values were means ± S.D. of three parallel measurements.

Crude carbohydrate, protein, and fat components of the 8 types of aquatic product matrix media were determined, and the results are presented in Figures 4A–C. Carbohydrate contents of the 8-matrix media ranged from 0.17 to 0.83‰, among which the highest one was *P. undulata* (0.83‰), followed by *M. quadrangularis Deshayes* (0.82‰) matrix. Conversely, the carbohydrate content of *C. idellus* matrix medium was the lowest (0.17‰). In addition, in the 4 types of fish matrix media, *A. nobilis* matrix contained higher carbohydrate content (0.40‰) than the 3 others (0.17–0.29‰); in the 4 types of shellfish matrix media, *P. undulata* matrix had the highest carbohydrate content (0.83‰), whereas *P. viridis* matrix the lowest (0.29‰) (Figure 4A).

As shown in Figure 4B, protein concentrations of the 8-matrix media ranged from 14.66 to 31.49‰, among which the highest one was *P. pekinensis* matrix (31.49‰), followed by *C. idellus* (24.38‰), and *M. quadrangularis Deshayes* (23.67‰). Conversely, the protein content of *M. antiquata* matrix was the lowest (14.66‰), followed by *P. undulata* (14.74‰), and *A. nobilis* (20.14‰).

As shown in Figure 4C, notably, the fat content of *M. quadrangularis Deshayes* matrix was also the highest (0.61‰), whereas *C. idellus* matrix was the lowest (0.27‰) among the 8 types of aquatic product matrix media.

### Survival of the *V. cholerae* Isolates in Diverse Aquatic Product Matrix Media

**Figure 5:**
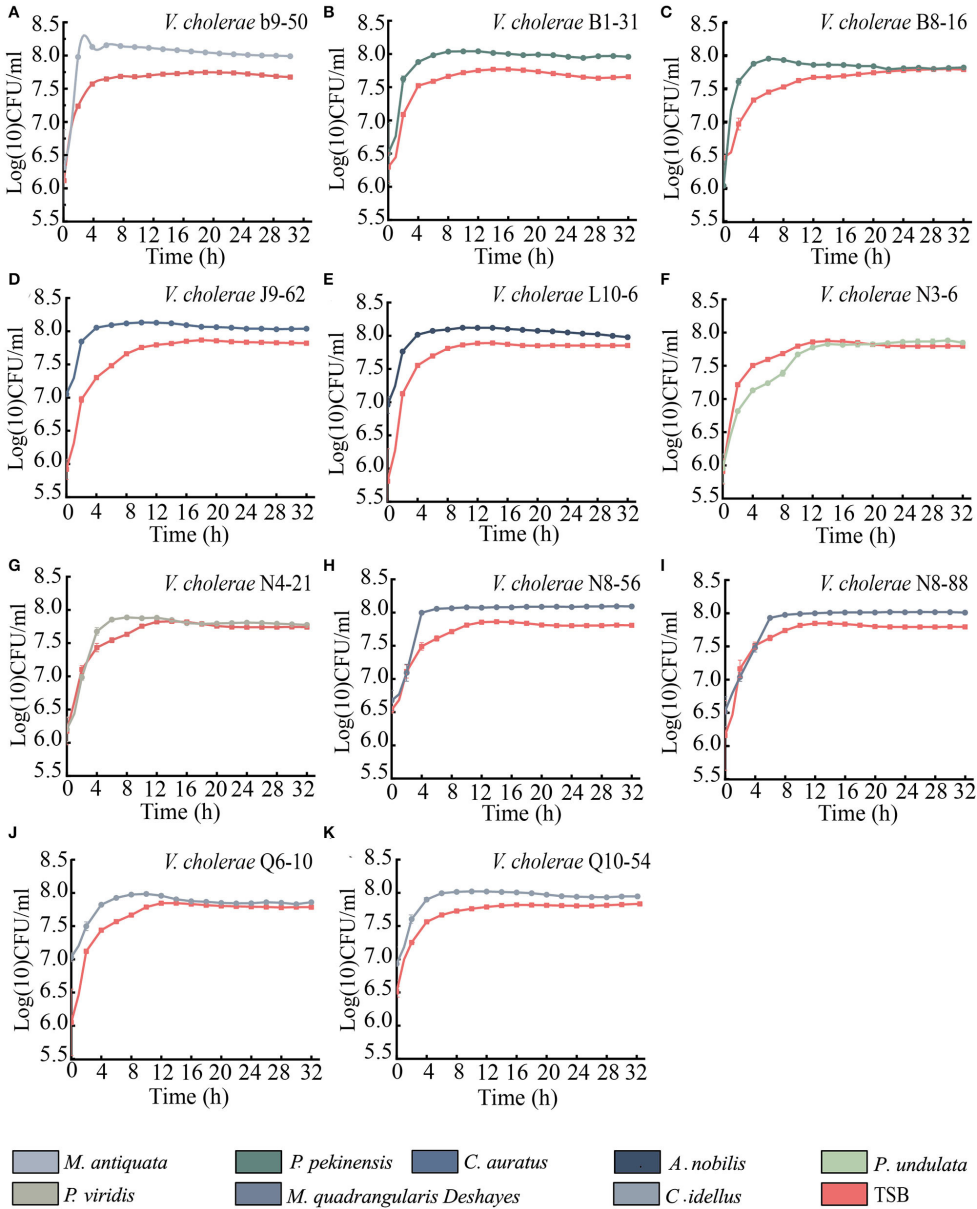
Survival of the 11 *V. cholerae* isolates incubated in diverse aquatic product matrices and TSB medium at 37°C. **(A–K)** *V. cholerae* b9-50, B1-31, B8-16, J9-62, L10-6, N3-6, N4-21, N8-56, N8-88, Q6-10, and Q10-54 isolates, respectively.

As shown in Figures 5A–K, when compared with the routine TSB medium, the maximum biomass of 9 *V. cholerae* isolates (b9-50, J9-62, N8-56, N8-88, B1-31, B8-16, L10-6, Q6-10, and Q10-54) were extremely higher at the stationary phase (SP) when incubated in *M. antiquata, C. auratus, M. quadrangularis Deshayes, P. pekinensis, A. nobilis*, and *C. idellus* matrix media (*p* < 0.01) (Figures 5A–E,H–K). For example, the maximum biomass of *V. cholerae* b9-50 in *M. antiquata* matrix was ~1-fold higher at Log (10) colony forming unit (CFU)/mL than that in the TSB medium (Figure 5A). Similarly, the growth of *V. cholerae* N4-21 isolate in *P. viridis* matrix was also significantly vigorous compared with the TSB medium (*p* < 0.05) (Figure 5G). Additionally, the growth of *V. cholerae* N3-6 isolate in *P. undulata* matrix showed no significant difference from that in the TSB medium (*p* > 0.05) (Figure 5F).

### Distinct Secretomes of the *V. cholerae* Isolates Grown in Diverse Aquatic Product Matrices

**Figure 6:**
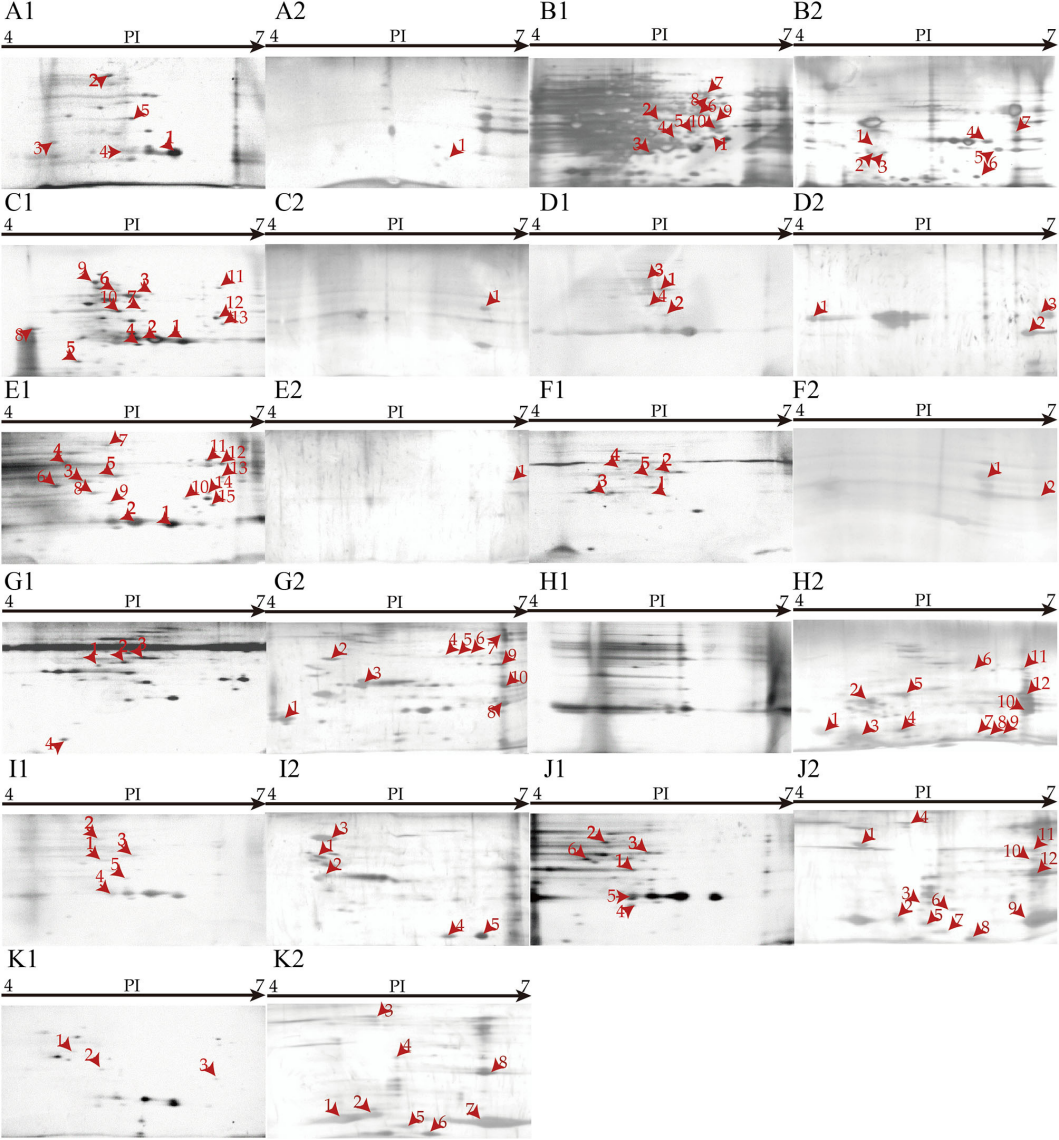
Secretomic profiles of the 11 *V. cholerae* isolates incubated in diverse aquatic product matrices and TSB medium at 37°C. **(A–K)** *V. cholerae* B1-31, B8-16, J9-62, L10-6, Q6-10, Q10-54, b9-50, N3-6, N4-21, N8-56, and N8-88 isolates were grown in the TSB medium at 37°C, respectively. **(A2–K2)** The *V. cholerae* isolates were grown in *P. pekinensis, C. auratus, A. nobilis, C. idellus, M. antiquata, P. undulata, P. viridis, and M. quadrangularis Deshayes* matrix media at 37°C, respectively.

Secretomes of the eleven *V. cholerae* isolates that were incubated in the eight types of aquatic product matrices were obtained by 2D-GE analysis (Figures 6A–K). Secretomic patterns produced by 3 independent 2D-GE experiments of each isolate were consistent (Figures not shown). Comparative secretomic analysis revealed 74 differential extracellular proteins (marked with different red numbers, Figure 6), which were secreted by the *V. cholerae* isolates when grown in the matrices and TSB medium, respectively. Amino acid sequences of each of these extracellular proteins were further determined by LC-MS/MS analysis.

Remarkably, *V. cholerae* b9-50, N3-6, N8-56, and N8-88 isolates, when grown in *M. antiquata, P. undulata*, and *M. quadrangularis Deshayes* (N8-56 and N8-88 isolates) matrices, respectively, secreted more extracellular proteins (n = 5 to 12) than those in the TSB medium. For example, *V. cholerae* N3-6 in *P. undulata* matrix secreted 12 more extracellular proteins than in the TSB medium. Conversely, *V. cholerae* B1-31, B8-16, J9-62, L10-6, Q6-10, and Q10-54 isolates, when incubated in *P. pekinensis* (B1-31 and B8-16 isolates), *C. auratus, A. nobilis, and C. idellus* (Q6-10 and Q10-54 isolates) matrices, respectively, secreted fewer extracellular proteins than those in the TSB medium (*n* = 1–14).

### Identification of Extracellular Proteins Secreted by the *V. cholerae* Isolates Grown in Diverse Aquatic Product Matrices

**Figure 7:**
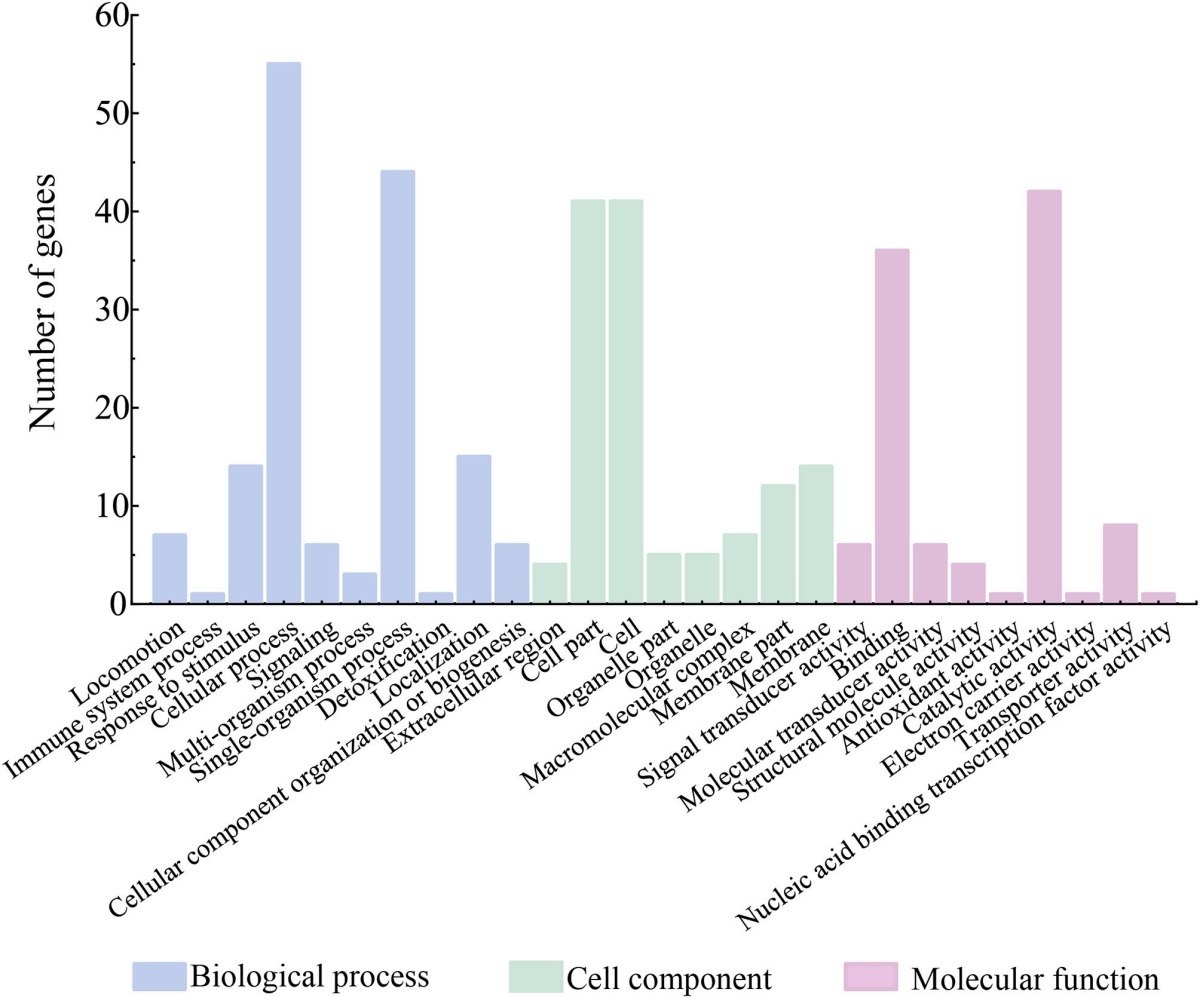
Gene functional classification of differential extracellular proteins secreted by the *V. cholerae* isolates.

Among the 74 identified extracellular proteins, 71 were classified into three main GO categories, including biological processes, cellular components, and molecular functions (Figure 7). Given that multiple biological functions can be assigned to a single identified protein, the most abundant GO function was the cellular process (77.46%, 55/71), followed by single-organism process (61.97%, 44/71), and catalytic activity (59.15%, 42/71). In contrast, the immune system process (1.41%, 1/71), detoxification (1.41%, 1/71), nucleic acid binding transcription factor activity (1.41%, 1/71), electron carrier activity (1.41%, 1/71), and antioxidant activity (1.41%, 1/71) showed the opposite patterns (Figure 7). Additionally, six identified extracellular proteins had no hit against the GO database.

### Effects of Diverse Aquatic Product Matrices on Secretomes of the *V. cholerae* Isolates

#### Effects of the Shellfish Matrices

The shellfish matrices highly increased extracellular protein secretion of *V. cholerae* b9-50, N3-6, N4-21, N8-56, and N8-88 isolates when grown in *M. antiquata, P. undulata, P. viridis*, and *M. quadrangularis Deshayes* (N8-56 and N8-88 isolates), respectively (Figure 6G2–K2).

For example, ~12 extracellular proteins were secreted by *V. cholerae* N3-6 when incubated in *P. undulata* matrix, including an antitoxin (Spot H2-1), a (ribosomal protein S18)-alanine N-acetyltransferase (Spot H2-6), a beta-lactamase family protein (Spot H2-7), a DNA-binding transcriptional regulator KdgR (Spot H2-9), a T2SS GspH family protein (Spot H2-10), an AAA family ATPase (Spot H2-11), a restriction endonuclease S subunit (Spot H2-12), and five unknown proteins (Spots H2-2, H2-3, H2-4, H2-5, and H2-8) (Figure 6H2). Notably, no extracellular protein was observed when *V. cholerae* N3-6 grown in the TSB medium (Figure 6H1).

Approximately 18 differential extracellular proteins were identified from secretomic profiles of *V. cholerae* N8-56 when incubated in *M. quadrangularis Deshayes* matrix and TSB medium, respectively. Among these, 12 extracellular proteins were secreted by *V. cholerae* N8-56 in *M. quadrangularis Deshayes* matrix, including an antitoxin (Spot J2-1), a (ribosomal protein S18)-alanine N-acetyltransferase (Spot J2-3), a beta-lactamase family protein (Spot J2-4), a malate dehydrogenase (Spot J2-5), an acetyltransferase (Spot J2-6), a copper homeostasis protein CutC (Spot J2-7), a transposase A (Spot J2-8), an ATP synthase subunit beta (Spot J2-9), a T2SS GspH family protein (Spot J2-10), a glycosyltransferase (Spot J2-11), a Type I restriction enzyme R protein (Spot J2-12), and an uncharacterized protein (Spot J2-2) (Figure 6J2). The other 6 extracellular proteins were secreted by *V. cholerae* N8-56 in the TSB medium, including a flagellar hook-associated protein FlgL (Spot J1-1), a chaperone protein DnaK (Spot J1-2), a bifunctional phosphoribosyl laminoimidazole carboxamide formyltransferase (Spot J1-3), an autonomous glycyl radical cofactor (Spot J1-4), a capsular polysaccharide synthesis CpsC (Spot J1-5), and an arginine ABC transporter (Spot J1-6) (Figure 6J1).

Approximately 14 differential extracellular proteins were identified from secretomic profiles of *V. cholerae* b9-50 grown in *M. antiquata* matrix and TSB medium, respectively. Among these, 10 extracellular proteins were secreted by *V. cholerae* b9-50 in *M. antiquata* matrix, including a chaperone protein DnaK (HSP70) (Spot G2-2), a GTP 3',8-cyclase (Spot G2-3), an aromatic amino acid aminotransferase (Spot G2-4), an AAA family ATPase (Spot G2-5), a DUF4435 domain-containing protein (Spot G2-6), a histidine kinase (Spot G2-7), a DNA sulfur modification protein DndD (Spot G2-8), a restriction endonuclease S subunit (Spot G2-9), an icmf-related protein (Spot G2-10), and an uncharacterized protein (Spot G2-1) (Figure 6G2). The other 4 extracellular proteins were secreted by *V. cholerae* b9-50 in the TSB medium, including a flagellar hook-associated protein FlgL (Spot G1-1), a bifunctional phosphoribosyl laminoimidazole carboxamide formyltransferase (Spot G1-2), a phosphoenolpyruvate carboxy kinase (Spot G1-3), and a thioredoxin reductase (Spot G1-4) (Figure 6G1).

Similarly, ~8 differential proteins were secreted by *V. cholerae* N8-88 when grown in *M. quadrangularis Deshayes* matrix (Figure 6K2), and 3 others in the TSB medium (Figure 6K1). *V. cholerae* N4-21 secreted 5, and 5 extracellular proteins in *P. viridis* matrix (Figure 6I2), and TSB medium (Figure 6I1), respectively.

#### Effects of the Fish Matrices

The fish matrix media significantly reduced the numbers of extracellular proteins secreted by *V. cholerae* J9-62, L10-6, Q6-10 and Q10-54, B1-31 and B8-16 isolates when cultured in *C. auratus, A. nobilis, C. idellus*, and *P. pekinensis* metrices (Figure 6A2–F2).

For instance, approximately 17 differential extracellular proteins were identified from secretomic profiles of *V. cholerae* B8-16 when incubated in *P. pekinensis* matrix and TSB medium, respectively. Among these, 7 extracellular proteins were secreted by *V. cholerae* B8-16 in *P. pekinensis* matrix (Figure 6B2), including a ribosomal RNA large subunit methyltransferase I (Spot B2-1), a ribosomal-protein-alanine N-acetyltransferase (Spot B2-2), a basal-body rod modified protein FlgD (Spot B2-3), a CinA family protein (Spot B2-4), a dehalogenase/epoxide hydrolase (Spot B2-5), a uridine phosphorylase (Spot B2-6), and a DUF4435 domain-containing protein (Spot B2-7) (Figure 6B2). The other 10 extracellular proteins were secreted by *V. cholerae* B8-16 in the TSB medium, including a succinate-CoA ligase (ADP-forming) subunit alpha (Spot B1-1), a transaldolase (Spot B1-2), a glyceraldey-3-phosphate dehydrogenase (Spot B1-3), a beta-lactamase (Spot B1-4), a methionyl-tRNA formyltransferase (Spot B1-5), an alanine dehydrogenase (Spot B1-6), an oligopeptide ABC transporter (Spot B1-7), a S8 family peptidase (Spot B1-8), a sulfate ABC transporter substrate-binding protein (Spot B1-9), and a TRAP transporter substrate-binding protein (Spot B1-10) (Figure 6B1).

Approximately one restriction endonuclease S subunit (Spot C2-1) was secreted by *V. cholerae* J9-62 when grown in *C. auratus* matrix (Figure 6C2), whereas 13 extracellular proteins were secreted in the TSB medium, including a phosphate transcriptional regulatory protein PhoB (Spot C1-1), a transaldolase (Spot C1-3), a glyceraldehyde-3-phosphate dehydrogenase (Spot C1-4), an RRf2-linked NADH-flavin reductase (Spot C1-5), a trigger factor (Spot C1-6), a beta-lactamase (Spot C1-7), an ompU (Spot C1-8), a deoxyribose-phosphate aldolase (Spot C1-9), a chaperone protein DnaK (Spot C1-10), a cysteine synthase (Spot C1-11), a NAD-dependent malic enzyme (Spot C1-12), an oligopeptide ABC transporter (Spot C1-13), and an uncharacterized protein (Spot C1-2) (Figure 6C1).

Approximately 7 differential extracellular proteins were identified from secretomic profiles of *V. cholerae* L10-6 grown in *A. nobilis* matrix and TSB medium, respectively. Among these, 3 extracellular proteins were secreted in *A. nobilis* matrix, including a flagellin (Spot D2-1), a (ribosomal protein S18)-alanine N-acetyltransferase (Spot D2-2), and a T2SS GspH family protein (Spot D2-3) (Figure 6D2). The other 4 extracellular proteins were secreted by *V. cholerae* L10-6 in the TSB medium, including a phosphoenolpyruvate carboxykinase (Spot D1-1), a beta-lactamase (Spot D1-2), a bifunctional phosphoribosyl laminoimidazole carboxamide formyltransferase (Spot D1-3), and a hemagglutinin/proteinase (Spot D1-4) (Figure 6D1).

Similarly, approximately two extracellular proteins were secreted by *V. cholerae* Q10-54 grown in *C. idellus* matrix (Figure 6F2), and five in the TSB medium (Figure 6F1); one extracellular protein was secreted by *V. cholerae* B1-31 in *P. pekinensis* matrix (Figure 6A2), and five in the TSB medium (Figure 6A1); one was secreted by *V. cholerae* Q6-10 in *C. idellus* matrix (Figure 6E2), and 15 in the TSB medium (Figure 6E1).

### Common Extracellular Proteins Secreted by the *V. cholerae* Isolates Grown in Diverse Aquatic Product Matrices

Among the 74 identified extracellular proteins, 5 were shared by some *V. cholerae* isolates grown in the aquatic product matrices, including a DUF4435 domain-containing protein (Spots B2-7, and G2-6), a restriction endonuclease S subunit (Spots C2-1, G2-9, and H2-12), a (ribosomal protein S18)-alanine N-acetyltransferase (Spots D2-2, H2-6, and J2-3), a T2SS-related protein (Spots D2-3, H2-10, J2-10, and K2-6), and a transposase A (Spots E2-1, F2-1, and J2-8) (Table 2). They were also common extracellular proteins secreted by the *V. cholerae* isolates when incubated in the fish matrices.

**Table 2:** Common extracellular proteins secreted by the *V. cholerae* isolates grown in diverse aquatic product matrices.

**Protein spot No**.	**Uniprot No**.	**Protein**	**Gene**	**Sequence coverage (%)**	**MW (Da)**	**PI**	**Putative function**	***V. cholerae* isolate**	**Matrix medium**
B2-7, G2-6	A0A5C9HLL1	DUF4435 domain-containing protein	*FXF03_10765*	1.66	34,867.78	6.90	–*	B8-16, b9-50	*P. pekinensis, M. antiquata*
C2-1, G2-9, H2-12	D7HH59	Restriction endonuclease S subunit	*VCRC385_00007*	1.14	49,618.55	7.02	DNA binding endonuclease activity, DNA modification, endonuclease, hydrolase	J9-62, b9-50, N3-6	*C. auratus, M. antiquata, P. undulata*
D2-2, H2-6, J2-3	A0A544I569	[Ribosomal protein S18]-alanine N-acetyltransferase	*rimI*	5.59	18,354.75	6.96	Acetylates the N-terminal alanine of ribosomal protein S18	L10-6, N3-6, N8-56	*A. nobilis, P. undulata, M. quadrangularis Deshayes*
D2-3, H2-10, J2-10, K2-6	A0A7X4TA57	T2SS GspH family protein	*FKR41_09995*	2.34	27,737.67	7.76	–*	L10-6, N3-6, N8-56, N8-88	*A. nobilis, P. undulata, M. quadrangularis Deshayes, M. quadrangularis* *Deshayes*
E2-1, F2-1, J2-8	A0A0D5XRR8	Transposase A	*tnpA*	2.84	36,164.99	7.58	Involved in the transposition of the insertion sequence IS5	Q6-10, Q10-54, N8-56	*C. idellus, C. idellus, M. quadrangularis* *Deshayes*
H2-1, J2-1, K2-1	A0A544BM56	Antitoxin	*FLM02_20230*	14.12	9,662.01	5.87	Antitoxin component of a type II toxin-antitoxin (TA) system	N3-6, N8-56, N8-88	*P. undulata, M. quadrangularis Deshayes, M. quadrangularis* *Deshayes*
H2-3, I2-1, K2-3	A0A5Q6PCL1	Uncharacterized protein	*F0M16_21990*	7.53	10,151.54	6.40	–*	N3-6, N4-21, N8-88	*P. undulata, P. viridis, M. quadrangularis* *Deshayes*
H2-5, I2-2, J2-2, K2-4	A0A655VTX7	Uncharacterized protein	*ERS013193_01478*	5.75	10,391.86	6.17	–*	N3-6, N4-21, N8-56, N8-88	*P. undulata, P. viridis, M. quadrangularis Deshayes, M. quadrangularis* *Deshayes*
H2-7, J2-4, K2-5	A0A6M6J0P0	Beta-lactamase family protein	*HND97_11440*	5.56	16,567.75	5.11	–*	N3-6, N8-56, N8-88	*P. undulata, M. quadrangularis Deshayes, M. quadrangularis* *Deshayes*
H2-8, I2-3	A0A5Q6PIT6	Uncharacterized protein	*F0M16_11010*	4.17	30,009.37	4.92	–*	N3-6, N4-21	*P. undulata, P. viridis*
I2-4, J2-9	A5F459	ATP synthase subunit beta	*atpD*	2.36	50,520.07	4.82	Produces ATP from ADP in the presence of a proton gradient across the membrane. The catalytic sites are hosted primarily by the beta subunits.	N4-21, N8-56	*P. viridis, M. quadrangularis* *Deshayes*

–* *, not detected*.

Similarly, there were 6 extracellular proteins shared by some *V. cholerae* isolates when incubated in the shellfish matrices, including an antitoxin (Spots H2-1, J2-1, and K2-1), a beta-lactamase family protein (Spots H2-7, J2-4, and K2-5), an ATP synthase subunit beta (Spots I2-4, and J2-9), and 3 uncharacterized proteins (Spots H2-3, I2-1, K2-3, H2-5, I2-2, J2-2, K2-4, H2-8, and I2-3) (Table 2).

### Identification of Common and Differential Intracellular Proteins of the *V. cholerae* Isolates Grown in Diverse Aquatic Product Matrices

A total of 8,119 intracellular proteins were identified from the eleven *V. cholerae* isolates when grown in the eight types of aquatic product matrix media by LC-MS/MS analysis. Comparative proteomic analyses revealed approximately 209 common intracellular proteins shared among the isolates, approximately 160 of which were classified into three major GO categories (Supplementary Figure S1A). Given that multiple biological functions could be assigned for single identified protein, the metabolic processes were most abundant (84.38%, 135/160), followed by cellular processes (82.50%, 132/160), and catalytic activity (75.63%, 121/160). Conversely, intracellular proteins in the developmental process (0.63%, 1/160), reproduction (0.63%, 1/160), and reproductive process (0.63%, 1/160) showed the opposite patterns (Supplementary Figure S1A).

Comparative proteomic analyses also revealed ~1,788 differential intracellular proteins among the *V. cholerae* isolates when grown in the matrices. Of these, 629 proteins were functionally classified into GO categories, whereas 1,159 proteins were unknown function in the *V. cholerae* isolates. The most abundant GO term of the differential intracellular proteins was the cellular process (81.08%, 510/629), followed by metabolic processes (78.06%, 491/629), and catalytic activity (70.27%, 442/629). The opposite patterns were membrane-enclosed lumen (0.16%, 1/629), reproduction (0.32%, 2/629), and reproductive process (0.32%, 2/629) (Supplementary Figure S1B).

#### Identification of Common and Differential Intracellular Proteins of the *V. cholerae* Isolates Grown in Fish Matrices

There were approximately 142 intracellular proteins shared by 6 *V. cholerae* isolates (B1-31, B8-16, J9-62, L10-6, Q6-10, and Q10-54) of the fish origins when grown in the fish *P. pekinensis, C. auratus, A. nobilis*, and *C. idellus* matrices, twelve of which showed no match against the GO database. Functional classification of the other 130 common intracellular proteins into GO categories is shown in Supplementary Figure S2A.

In addition, there were ~861 differential intracellular proteins produced by the 6 *V. cholerae* isolates grown in the fish matrices. Remarkably, approximately 457 of these proteins were unknown functions. Among the other 404 differential intracellular proteins, the most abundant category was the cellular process (84.65%, 342/404), followed by metabolic process (79.70%, 322/404), and catalytic activity (74.26%, 300/404) (Supplementary Figure S2B).

#### Identification of Common and Differential Intracellular Proteins in the *V. cholerae* Isolates Grown in Shellfish Matrices

Comparative proteomic analyses also revealed ~229 intracellular proteins shared by 5 *V. cholerae* isolates (b9-50, N3-6, N4-21, N8-56, and N8-88) of the shellfish origins when grown in the shellfish *M. antiquata, P. undulata, P. viridis*, and *M. quadrangularis Deshayes* matrices. Approximately 31 of these proteins showed no match against the GO database. The most abundant GO category of the other 198 common proteins was the metabolic process (81.82%, 162/198), followed by cellular process (79.29%, 157/198), and catalytic activity (72.22%, 143/198) (Supplementary Figure S3A).

Similarly, there were 979 differential intracellular proteins produced by the 5 *V. cholerae* isolates grown in the shellfish matrices, approximately 469 of which showed no match against the GO database. The other 510 differential intracellular proteins were grouped into GO categories (Supplementary Figure S3B).

### Virulence-Associated Proteins of the *V. cholerae* Isolates Grown in Diverse Aquatic Product Matrices

#### Extracellular Virulence-Associated Proteins

Bacterial virulence is ‘the ability to enter, replicate within, and persist at host sites that are inaccessible to commensal species' (Falkow, 1991). Among the 74 identified extracellular proteins, several were involved in invasion, damage of host cells and tissues, or adhesion of pathogenic bacteria (Supplementary Table 3), e.g., an icmF-related protein (Spots G2-10), a T2SS GspH family protein (Spots D2-3, H2-10, J2-10, and K2-6), and a flagellin (Spots D2-1). Notably, *V. cholerae* b9-50 grown in *M. antiquata* matrix secreted more extracellular virulence-associated proteins (*n* = 3) than the other isolates. No such protein was found in secretomic profiles derived from *V. cholerae* B1-31, J9-62, Q6-10, and Q10-54 isolates when grown in *M. antiquata, C. auratus*, and *C. idellus* matrices.

#### Intracellular Virulence-Associated Proteins

Among the 8,119 identified intracellular proteins, approximately 83 were directly or indirectly involved in the virulence of pathogenic bacteria, and those referred to adhesion, invasion, damage of host cells and tissues, and regulation of virulence are shown in Supplementary Table 3. Of these, ~41 were produced by 5 *V. cholerae* isolates (b9-50, N3-6, N4-21, N8-56, and N8-88) when cultured in the 4 types of shellfish matrices, e.g., a T6SS tip protein VgrG (Spots S592), a chemotaxis protein CheA (Spots S593), an autoinducer 2-binding periplasmic protein LuxP (Spots S596), a transcription elongation factor GreA (Spots S599), a phosphoglycerate kinase (Spots S606), and an Hcp family T6SS effector (Spots S621). Among these virulence-associated proteins, 9 were shared by the 5 *V. cholerae* of the shellfish origins, including an oligopeptide ABC transporter substrate-binding protein OppA (Spots s16; b9-50 and N3-6 isolates), an UTP-glucose-1-phosphate uridylyltransferase (Spots s53; b9-50 and N8-88 isolates), an endoribonuclease L-PSP (Spots s55; b9-50 and N4-21 isolates), an oligopeptide ABC transporter substrate-binding protein OppA (Spots s108; N4-21, N8-56, and N8-88 isolates), a pantothenate synthetase (PS) (Spots s146; N4-21, N8-56, and N8-88 isolates), a maltoporin (Spots s191; N4-21 and N8-56 isolates), a HlyD family secretion protein (Spots s217; N8-56 and N8-88 isolates), and two 50 S ribosomal protein L6 (Spots s34, and s219; b9-50, N3-6, N4-21, N8-56 and N8-88 isolates). Remarkably, *V. cholerae* N4-21 grown in *P. viridis* matrix produced the highest number of intracellular virulence-associated proteins (*n* = 36), consisted with the higher number of predicted virulence genes (*n* = 525) in its genome, whereas *V. cholerae* N3-6 grown in *P. undulata* matrix produced the fewest (*n* = 4).

Similarly, 38 intracellular virulence-associated proteins were produced by 6 *V. cholerae* isolates (B1-31, B8-16, J9-62, L10-6, Q6-10, and Q10-54) when incubated in the 4 types of fish matrices, e.g., a cyclic AMP receptor protein (Spots F263), a Tol-Pal system protein TolB (Spots F292), a protein-export protein SecB (Spots F491), a trigger factor (Spots F719), and a T1SS TolC (Spots F721) (Supplementary Table 3). Of these, 3 were shared by the isolates of the fish origins, including a peptidoglycan-associated lipoprotein (Spots f37; B1-31 and B8-16 isolates), a lactoylglutathione lyase (Spots f50; B1-31 and Q6-10 isolates), and a peptide deformylase (PDF) (Spots f51; B1-31 and J9-62 isolates). Notably, *V. cholerae* B8-16 grown in *P. pekinensis* matrix produced the maximum number of intracellular virulence-associated proteins (*n* = 33), consisted with higher predicted virulence genes (*n* = 536) in its genome. Conversely, *V. cholerae* L10-6 grown in *A. nobilis* matrix produced the minimum of such proteins (*n* = 6).

Additionally, a few intracellular virulence-associated proteins were shared by some *V. cholerae* isolates when incubated in the shellfish or fish matrices, including a 50S ribosomal subunit assembly factor BipA (Spots S119, S445, S797, F190, and F380), a glyceraldehyde-3-phosphate dehydrogenase (Spots S299, S573, S742, S898, F2, F54, F220, F652, and F709), and a Tol-Pal system protein TolB (Spots S443, S850, F292, and F691) (Supplementary Table 3).

### Antibiotic Resistance-Associated Proteins of the *V. cholerae* Isolates Grown in Diverse Aquatic Product Matrices

#### Extracellular Resistance-Associated Proteins

Approximately 4 extracellular proteins involved in bacterial resistance were secreted by *V. cholerae* B8-16, N3-6, N8-56, and N8-88 isolates when grown in *P. pekinensis, P. undulata*, and *M. quadrangularis Deshayes* (N8-88 and N8-56 isolates), respectively. They included a ribosomal RNA large subunit methyltransferase I (Spots B2-1), a ribosomal-protein-alanine N-acetyltransferase (Spots B2-2), an antitoxin (Spots H2-1, J2-1, and K2-1), and a beta-lactamase family protein (Spots H2-7, J2-4, and K2-5) (Table 3).

**Table 3:** Putative extracellular and intracellular resistance-associated proteins secreted and produced by the *V. cholerae* isolates grown in diverse aquatic product matrices.

**Protein spot no**.	**Uniprot no**.	**Protein**	**Gene**	**Sequence coverage (%)**	**MW (Da)**	**PI**	**Putative function**	***V. cholerae* isolate**	**Matrix**	**References**
**Putative extracellular resistance-associated proteins**										
B2-1	A0A655P0E0	Ribosomal RNA large subunit methyltransferase I	*rlmI*	7.84	16,983.29	5.15	Methyltransferase, transferase S-adenosyl-L-methionine	B8-16	*P. pekinensis*	Takamatsu et al., 2018
B2-2	A0A7X4PHW6	Ribosomal-protein-alanine N-acetyltransferase	*rimI*	5.59	18,293.56	6.18	-*	B8-16	*P. pekinensis*	Zheng et al., 2021
H2-1, J2-1, K2-1	A0A544BM56	Antitoxin	*FLM02_20230*	14.12	9,662.01	5.87	Antitoxin component of a type II toxin-antitoxin (TA) system	N3-6, N8-56, N8-88	*P. undulata, M. quadrangularis Deshayes, M. quadrangularis Deshayes*	Harms et al., 2018
H2-7, J2-4, K2-5	A0A6M6J0P0	Beta-lactamase family protein	*HND97_11440*	5.56	16,567.75	5.11	–*	N3-6, N8-56, N8-88	*P. undulata, M. quadrangularis Deshayes, M. quadrangularis Deshayes*	White et al., 2017
**Putative intracellular resistance-associated proteins**										
T119	C3LSH1	50S ribosomal subunit assembly factor BipA	*typA*	14.78	67,184.73	5.15	Hydrolase, rRNA-binding, tRNA-binding, ribosome biogenesis, GTP-binding, nucleotide-binding	b9-50	*M. antiquata*	Gibbs and Fredrick, 2018
D190	A0A0H3Q4Q0	50S ribosomal subunit assembly factor BipA	*bipA*	13.03	67,770.44	5.19	Hydrolase, rRNA-binding, tRNA-binding, ribosome biogenesis, GTP-binding, nucleotide-binding	B8-16	*P. pekinensis*	Goh et al., 2021
D380	A0A0H3AMD9	50S ribosomal subunit assembly factor BipA	*typA*	19.38	67,184.73	5.15	Hydrolase, rRNA-binding, tRNA-binding, ribosome biogenesis, GTP-binding, nucleotide-binding	J9-62	*C. auratus*	Goh et al., 2021
T445	A0A0H6M3P6	50S ribosomal subunit assembly factor BipA	*typA*	24.96	67,184.73	5.15	Hydrolase, rRNA-binding, tRNA-binding, ribosome biogenesis, GTP-binding, nucleotide-binding	N4-21	*P. viridis*	Gibbs and Fredrick, 2018
T797	A0A0H6M3P6	50S ribosomal subunit assembly factor BipA	*typA*	20.69	67,184.73	5.15	Hydrolase, rRNA-binding, tRNA-binding, ribosome biogenesis, GTP-binding, nucleotide-binding	N8-56	*M. quadrangularis Deshayes*	Gibbs and Fredrick, 2018
T250	A0A0H3Q0Q0	Outer membrane protein A	*VCE_003505*	16.82	34,285.06	5.07	Porin, ion transport, transport	b9-50	*M. antiquata*	Nie et al., 2020
T388	A0A5C2AX76	ABC transporter substrate-binding protein	*F0315_04640*	53.67	57,654.91	5.45	transmembrane transport	N4-21	*P. viridis*	Cassio Barreto De Oliveira and Balan, 2020
T490	A0A1X1LFL9	ABC transporter substrate-binding protein	*FKR41_00850 FXE88_12515*	39.24	37,677.27	6.25	–*	N4-21	*P. viridis*	Cassio Barreto De Oliveira and Balan, 2020
T549	A0A085Q5T2	Amino acid ABC transporter substrate-binding protein	*argT*	35.27	28,465.22	5.49	Amino-acid transport, transport	N4-21	*P. viridis*	Cassio Barreto De Oliveira and Balan, 2020
T446	A0A5C2B0D9	TolC family outer membrane protein	*F0315_11715*	32.72	48,555.57	4.97	Transport	N4-21	*P. viridis*	Pattanayak et al., 2021
T606	C3LRZ4	Phosphoglycerate kinase	*pgk*	25.00	41,564.35	4.96	Kinase, transferase, glycolysis, ATP-binding, nucleotide-binding	N4-21	*P. viridis*	Zhu et al., 2020
T750	A0A085TAV8	Phosphoglycerate kinase	*pgk*	74.94	40,978.63	4.91	Kinase, transferase, glycolysis, ATP-binding, nucleotide-binding	N8-56	*M. quadrangularis Deshayes*	Zhu et al., 2020
D205	C3LS33	Protein RecA	*recA*	20.63	45,183.07	5.72	DNA-binding, DNA damage, DNA recombination, DNA repair, SOS response, ATP-binding, nucleotide-binding	B8-16	*P. pekinensis*	Pavlopoulou, 2018
D540	A0A5B1BZP8	Signal peptide peptidase SppA	*sppA*	8.77	67,082.67	5.00	–*	J9-62	*C. auratus*	Henriques et al., 2020

–* *, not detected*.

#### Intracellular Resistance-Associated Proteins

Similarly, approximately 8 intracellular proteins involved in bacterial resistance were identified (Table 3). Of these, 6 were produced by *V. cholerae* b9-50, N4-21, and N8-56 isolates when grown in the shellfish *M. antiquata, P. viridis*, and *M. quadrangularis Deshayes* matrices, respectively, including a 50 S ribosomal subunit assembly factor BipA (Spots T119, T445, and T797), an Omp A (Spots T250), an ABC transporter substrate-binding protein (Spots T388, and T490), an amino acid ABC transporter substrate-binding protein (Spots T549), a phosphoglycerate kinase (Spots T606, and T750), and a TolC family outer membrane protein (Spots T446).

The *V. cholerae* B8-16, and J9-62 isolates grown in the fish *P. pekinnensis*, and *C. auratus* matrices, respectively, produced 3 resistance-associated proteins, including a protein RecA (Spots D205), a signal peptide peptidase SppA (Spots D540), and a 50S ribosomal subunit assembly factor BipA (Spots D190, and D380). The latter was also expressed by the *V. cholerae* isolates grown in the shellfish matrices.

Notably, *V. cholerae* N4-21 grown in *P. viridis* matrix produced the highest number of intracellular resistance-associated proteins (*n* = 5), whereas none was identified from *V. cholerae* N3-6, N8-88, B1-31, L10-6, Q6-10, and Q10-54 isolates when incubated in their corresponding matrices.

### Effects of Diverse Aquatic Product Matrices on Virulence- and Resistance-Associated Proteins Produced by the *V. cholerae* Isolates

Distinct secretomes and proteomes of the eleven *V. cholerae* isolates were induced by the eight types of aquatic animal matrices. When incubated in the TSB medium, only 7 virulence- and 5 resistance-associated extracellular proteins were secreted by these isolates (Shan et al., 2021). Nevertheless, when grown in the aquatic product matrices, 2 additional virulence-associated extracellular proteins were identified. Meanwhile, remarkably, there were 61 additional virulence-associated intracellular proteins were expressed by the *V. cholerae* isolates, which were absent from their proteomes derived from the TSB medium.

To confirm the identified proteins by 2D-GE and LC-MS/MS analyses, qRT-PCR assay was performed to detect the expression of randomly chosen differential proteins. The obtained data were generally consistent with those by the secretomic and proteomic analyses in this study (Supplementary Table S2).

## Discussion

The *V. cholerae* has been isolated from many species of aquatic animals (Xu et al., 2019; Chen et al., 2021). In China, freshwater fish production was 25,863,823 tons in 2020 (National Bureau of Statistics, http://www.stats.gov.cn/, accessed on 20 August 2021), and contributed importantly to China's fishery production, the top four famous species of which included *C. idellus, A. nobilis, C. auratus*, and *P. pekinensis*. The shellfish production was 14,800,800 tons in 2020 in China, and the main species included *M. antiquata, M. quadrangularis Deshayes, P. undulata*, and *P. viridis*. Identification of risk factors in *V. cholerae* of aquatic product origins is crucial for assuring food safety systems, particularly in developing nations (Chen and Alali, 2018). To the best of our knowledge, this study was the first to determine the survival of *V. cholerae* isolates in the commonly consumed fish and shellfish matrices. Our results revealed that the 8 types of fish and shellfish matrices (except *P. undulata*) highly increased the biomass of the eleven *V. cholerae* isolates, when compared with the routine TSB medium, indicating that the matrices benefited the persistence of *V. cholerae* in the edible aquatic animals.

Based on the obtained draft genomes in our previous research (Shan et al., 2021), comparative genomic analyses demonstrated considerable genome variation among the *V. cholerae* isolates. Approximately putative 524–559 virulence-related genes, and 186–207 resistance-related genes were predicted in the eleven *V. cholerae* genomes. Balakhonov et al. (2015) reported 7 virulence genes and 60 antibiotic resistance genes in the draft genome of *V. cholerae* El Tor O1 N16961. Verma et al. (2019) identified 117 virulence genes in *V. cholerae* IDH06781 genome. Compared with the previous reports (Balakhonov et al., 2015; Verma et al., 2019), in this study, the eleven *V. cholerae* isolates of aquatic animal origins carried more virulence- and resistance-associated genes, which was consistent with their resistance phenotypes to multiple antibiotic drugs and heavy metals (Shan et al., 2021).

In this study, comparative secretomic analyses revealed several extracellular virulence-associated proteins secreted by the *V. cholerae* isolates when grown in the aquatic product matrices, most of which were different from those in the TSB medium (Shan et al., 2021). Moreover, they were specifically secreted by the *V. cholerae* isolates in different matrices. For example, the T2SS-related GspH family protein (Spots D2-3, H2-10, J2-10, and K2-6) was secreted by *V. cholerae* L10-6, N3-6, N8-56, and N8-88 isolates when incubated in *A. nobilis, P. undulata, M. quadrangularis Deshayes*, and *M. quadrangularis Deshayes* matrices, respectively. This protein can enhance the adhesion and motility of *Pseudomonas aeruginosa* (Nguyen et al., 2015). In this study, another T2SS-related icmF protein (Spot G2-10) was secreted by *V. cholerae* b9-50 grown in *M. antiquata* matrix. It has been reported that the icmF was involved in motion, adhesion, and conjugation in *V. cholerae* (Zusman et al., 2004). The chaperone protein Dnak (Spot G2-2), secreted by *V. cholerae* b9-50 in *M. antiquata* matrix, played a critical role in maintaining intracellular protein homeostasis and protecting cells from toxic stress in *E. coli* (Zuiderweg et al., 2017; Zwirowski et al., 2017). The flagellin (Spot D2-1), secreted by *V. cholerae* L10-6 and Q10-54 in *A. nobilis* and *C. idellus* matrices, respectively, was a key subunit protein of flagella and was convinced as a virulence factor that contributes to host cell adhesion and invasion (Hajam et al., 2017). This virulence-related protein was also secreted by the *V. cholerae* isolates when cultured in TSB medium.

Interestingly, the antitoxins (Spots H2-1, J2-1, and K2-1) were secreted by some *V. cholerae* isolates when incubated in the shellfish matrices. In bacteria, toxin-antitoxin (T-A) systems can mediate cellular response to external stress by initiating processes, such as biofilm formation and programmed cell death (Álvarez et al., 2020). Recently, Janczak et al. reported that chromosomal localization of PemIK toxin-antitoxin system resulted in the loss of toxicity-characterization of pemIK (Sa1)-Sp from *Staphylococcus pseudintermedius*. The pemIK(Sa1)-Sp was homologous to the plasmid-encoded, highly toxic PemIKSa TA system in pathogenic *Staphylococcus aureus* (Janczak et al., 2020). The functionality of type II T-A systems of Gram-positive, strictly anaerobic, spore-forming pathogen *Clostridioides difficile* R20291 in human intestine has also been evaluated, of which MazEF and RelBE systems were functional in a heterologous expression system, and their corresponding toxins possessed an endoribonuclease activity (Álvarez et al., 2020).

In this study, comparative proteomic analyses revealed 83 putative intracellular virulence-associated proteins produced by the *V. cholerae* isolates when grown in diverse aquatic product matrices. Adhesion to host cells was necessary for pathogens to invade and persist in host epithelial and immune cells (Huang et al., 2019). In this study, adhesion-related virulence factors were produced by the *V. cholerae* isolates. For example, the OmpA (Spots S347), expressed by *V. cholerae* N3-6 in *P. undulata* matrix, was a key virulence factor mediating bacterial biofilm formation, eukaryotic cell infection, antibiotic resistance, and adherence to host cells in pathogenic *Acinetobacter baumannii* (Nie et al., 2020). Valeru et al. (2014) reported a regulating rule for OmpA in survival of *V. cholerae* and outer membrane vesicles as a potent virulence factor for this bacterium toward eukaryotes in the environment.

Bacterial chemotactic system is a typical coupling protein-dependent signal transduction system and plays a key role in bacterial colonization and pathogenicity (Huang et al., 2019; Korolik, 2019). In this study, chemotaxis proteins CheW (Spots S219) and CheY (Spots S221) were expressed by *V. cholerae* b9-50 grown in *M. antiquata* matrix; CheA (Spots S593) expressed by *V. cholerae* N4-21 in *P. viridis* matrix; and a 3'3'-CGAMP-specific phosphodiesterase 2 (Spots S629) expressed by *V. cholerae* N4-21 in *P. viridis* matrix. The latter was initially identified in the seventh epidemic *V. cholerae* and involved in effective intestinal colonization and chemotactic regulation (Deng et al., 2018).

Secretion systems-associated proteins were also expressed by the *V. cholerae* isolates when grown in diverse aquatic product matrices. For example, the T6SS-related VgrG (Spots S592, and S714) was expressed by *V. cholerae* N4-21 when grown in *P. viridis* matrix; Hcp (Spots S621, and S865) expressed by *V. cholerae* N4-21 and N8-56 isolates in *P. viridis* and *M. quadrangularis Deshayes* matrices, respectively; and icmF (Spots S887) produced by *V. cholerae* N8-56 in *M. quadrangularis Deshayes* matrix. The outer membrane protein TolC of T1SS (Spots F721), expressed by *V. cholerae* Q10-54 in *C. idellus* matrix, was involved in the transmission of virulence factors to host cells (Kopping et al., 2019). The Tol-Pal system protein TolB (Spots F292, F691, S443 and S850), expressed by *V. cholerae* B8-16, Q6-10, N4-21, and N8-56 in *P. pekinensis, C. idellus, P. viridis*, and *M. quadrangularis Deshayes* matrices, respectively, played a crucial role in bacterial adhesion and secretion (Hirakawa et al., 2020).

Virulence factors involved in invading and persisting in host cells were also identified in the *V. cholerae* isolates when incubated in the aquatic product matrices. For example, the DNA-binding protein HU (Spots S280), expressed by *V. cholerae* b9-50 in *M. antiquata* matrix, can regulate many cellular processes and the pathogenesis of bacteria, such as survival, stress response, virulence gene expression, and cell division (Martínez et al., 2015; Stojkova et al., 2019). The peptidase B, expressed by 6 *V. cholerae* isolates of the fish origins, was the main virulence factor in *Leishmania* spp. (Beyzay et al., 2017; Yao, 2020). The cyclic AMP receptor protein (Spots F263), expressed by *V. cholerae* B8-16 in *P. pekinensis* matrix, was an important transcription regulator of *Yersinia pestis* through direct or indirect mechanisms to regulate virulence and metal acquisition (Ritzert et al., 2019). Manneh-Roussel et al. (2018) reported that the cAMP receptor protein controled *V. cholerae* gene expression in response to host colonization. The hemolysin protein (Spots F527), expressed by *V. cholerae* J9-62 in *C. auratus* matrix, was considered as an important toxic factor of *S. aureus*, and *V. cholerae* toxicity (Liu et al., 2020; Wang et al., 2022).

Previous studies have indicated that virulence of marine pathogens was regulated by environmental stress conditions. For example, Zhou et al. recently revealed the role of pivotal virulence regulator ToxR in switching on the viable, but non-culturable state by sensing unfavorable environmental signals such as endogenous reactive oxygen species (hydrogen peroxide, H_2_O_2_) in *Vibrio alginolyticus*, which infects humans and aquatic animals causing severe economic losses (Zhou et al., 2022). Yin et al. (2021) provided evidence for putative roles of two (p)ppGpp synthetase genes (*relA* and *spoT*) attributing environmental adaption and virulence regulation in *V. alginolyticus*. A lipid II flippase MviN was found to mediate the regulation of environmental osmotic pressure on *esrB* of the EsrA-EsrB two-component system to control the virulence in the marine fish pathogen *Edwardsiella piscicida* (Yin et al., 2020b). Shao et al. also found that the interplay between ferric uptake regulator Fur and horizontally acquired virulence regulator EsrB coordinated virulence gene expression in *E. piscicida* (Shao et al., 2021). A leucyl aminopeptidase PepA was found to bind to and negatively regulate *esrB* to control virulence in *E. piscicida* (Yin et al., 2020a). Additionally, Buchad and Nair (2021) reported a new mechanism in which small RNA (sRNA) SprX modulated *S. aureus* pathogenicity by regulating the regulator WalR of autolysins (Buchad and Nair, 2021). In this study, the aquatic product matrices possibly mediated environmental nutritional and/or osmotic pressure changes, which induced extracellular and intracellular virulence-associated proteins secretion and production in the *V. cholerae* isolates.

In this study, comparative secretomic and proteomic analyses also revealed extracellular and intracellular resistance-associated proteins in the *V. cholerae* isolates when incubated in the aquatic product matrix media, indicating different molecular strategies, by which these isolates were resistant to antimicrobial drugs. For example, the beta-lactamase family protein (Spots H2-7, J2-4, and K2-5), secreted by *V. cholerae* N3-6, N8-56, and N8-88 isolates in *P. undulata*, and *M. quadrangularis Deshayes* matrices, played a key role in bacterial resistance to antibiotics (White et al., 2017). The ribosomal RNA large subunit methyltransferase I (Spots B2-1) was secreted by *V. cholerae* B8-16 in *P. pekinensis* matrix. Deletion of this methyltransferase I resulted in the increased susceptibility of *Melissococcus plutonius* to mirosamicin antibiotics (Takamatsu et al., 2018). On the other hand, the ABC transporter substrate binding protein (Spots T388, T490, and T549) was produced by *V. cholerae* N4-21 when grown in *P. viridis* matrix, which participated in cell detoxification, antibiotic, and drug efflux in *M. tuberculosis* and greatly affected the survival and development of many drug-resistant strains (Cassio Barreto De Oliveira and Balan, 2020). Moreover, the TolC family outer membrane protein (Spots T446), produced by *V. cholerae* N4-21 in *P. viridis* matrix, was present in many pathogenic Gram-negative bacteria, including *V. cholerae, E. coli*, and *P. aeruginosa*, and formed an outer membrane channel that removed drugs and toxins from cells (Leong et al., 2014; Pattanayak et al., 2021). The BipA (Spots T119, T445, T797, D190 and D380) was expressed by *V. cholerae* b9-50, N4-21 and N8-56, and B8-16 and J9-62 isolates, which played an important role for virulence, antimicrobial resistance, and biofilm formation in *P. aeruginosa* (Gibbs and Fredrick, 2018). The SppA (Spots D540), expressed by *V. cholerae* J9-62 in *C. auratus* matrix, was originally described as a signal peptide peptidase, and later shown to be resistant to lantibiotics (Henriques et al., 2020).

Overall, this study was the first to investigate the impact of commonly consumed aquatic animal matrices on the survival and pathogenicity of *V. cholerae* isolates. The growth of the *V. cholerae* isolates was highly enhanced when incubated in most matrices compared with the routine TSB medium. Distinct secretomes and proteomes of the eleven *V. cholerae* isolates were induced by diverse aquatic animal matrices. Comparative secretomic analyses revealed 74 differential extracellular proteins, including several putative virulence- and resistance-associated extracellular proteins in the *V. cholerae* isolates, when grown in the matrices. Meanwhile, comparative proteomic analyses revealed 83 intracellular virulence- and 8 intracellular resistance-associated proteins, of which 61 virulence-associated proteins were absent from proteomes of these isolates when grown in the TSB medium. Additionally, comparative genomic and proteomic analyses revealed a number of novel and strain-specific proteins with unknown function in the *V. cholerae* isolates. In future research, functions of these proteins together with the identified virulence- and resistance-associated proteins should be further investigated by cell and animal mode analysis. Taken together, the results in this study demonstrate that distinct secretomes and proteomes induced by the aquatic animal matrices facilitate *V. cholerae* persistence in the edible aquatic animals and enhance the pathogenicity of the leading waterborne pathogen worldwide.

## Data Availability Statement

The datasets presented in this study can be found in online repositories. The names of the repository/repositories and accession number(s) can be found in the article/Supplementary Material.

## Author Contributions

LY, YJ, XP, SQ, and LC participated in the design and/or discussion of the study. LY carried out the experiments and drafted the original manuscript. BZ and YX participated in the data analysis. LC revised the manuscript. All authors read and approved the final version to be published.

## Funding

This study was supported by the grants from Shanghai Municipal Science and Technology Commission (No. 17050502200) and National Natural Science Foundation of China (No. 31671946).

## Conflict of Interest

The authors declare that the research was conducted in the absence of any commercial or financial relationships that could be construed as a potential conflict of interest.

## Publisher's Note

All claims expressed in this article are solely those of the authors and do not necessarily represent those of their affiliated organizations, or those of the publisher, the editors and the reviewers. Any product that may be evaluated in this article, or claim that may be made by its manufacturer, is not guaranteed or endorsed by the publisher.

## Supplementary Material

Supplementary Figure S1 Gene functional classification of common and differential intracellular proteins produced by the 11 *V. cholerae* isolates grown in the eight types of aquatic product matrices. **(A)** Common proteins. **(B)** Differential proteins.

Supplementary Figure S2 Gene functional classification of common and differential intracellular proteins produced by 6 *V. cholerae* isolates grown in the four types of fish matrices. **(A)** Common proteins. **(B)** Differential proteins.

Supplementary Figure S3 Gene functional classification of common and differential intracellular proteins produced by 5 *V. cholerae* isolates grown in the four types of shellfish matrices. **(A)** Common proteins. **(B)** Differential proteins.

Supplementary Figure S4 The expression of several representative genes by the RT-PCR assay.

Supplementary Table S1 Genome features of the eleven *V. cholerae* isolates originating from eight species of aquatic animals.

Supplementary Table S2 The primers used in the qRT-PCR assay.

Supplementary Table S3 Putative extracellular and intracellular virulence-associated proteins secreted and produced by the V. cholerae isolates grown in diverse aquatic product matrices.
