# Supplementary figures and images for: Diverse Aquatic Animal Matrices Play a Key Role in Survival and Potential Virulence of Non-O1/O139 Vibrio cholerae Isolates
Source: Front Microbiol. 2022 Jun 21;13:896767. doi: 10.3389/fmicb.2022.896767 (PMC9255913; doi:10.3389/fmicb.2022.896767)

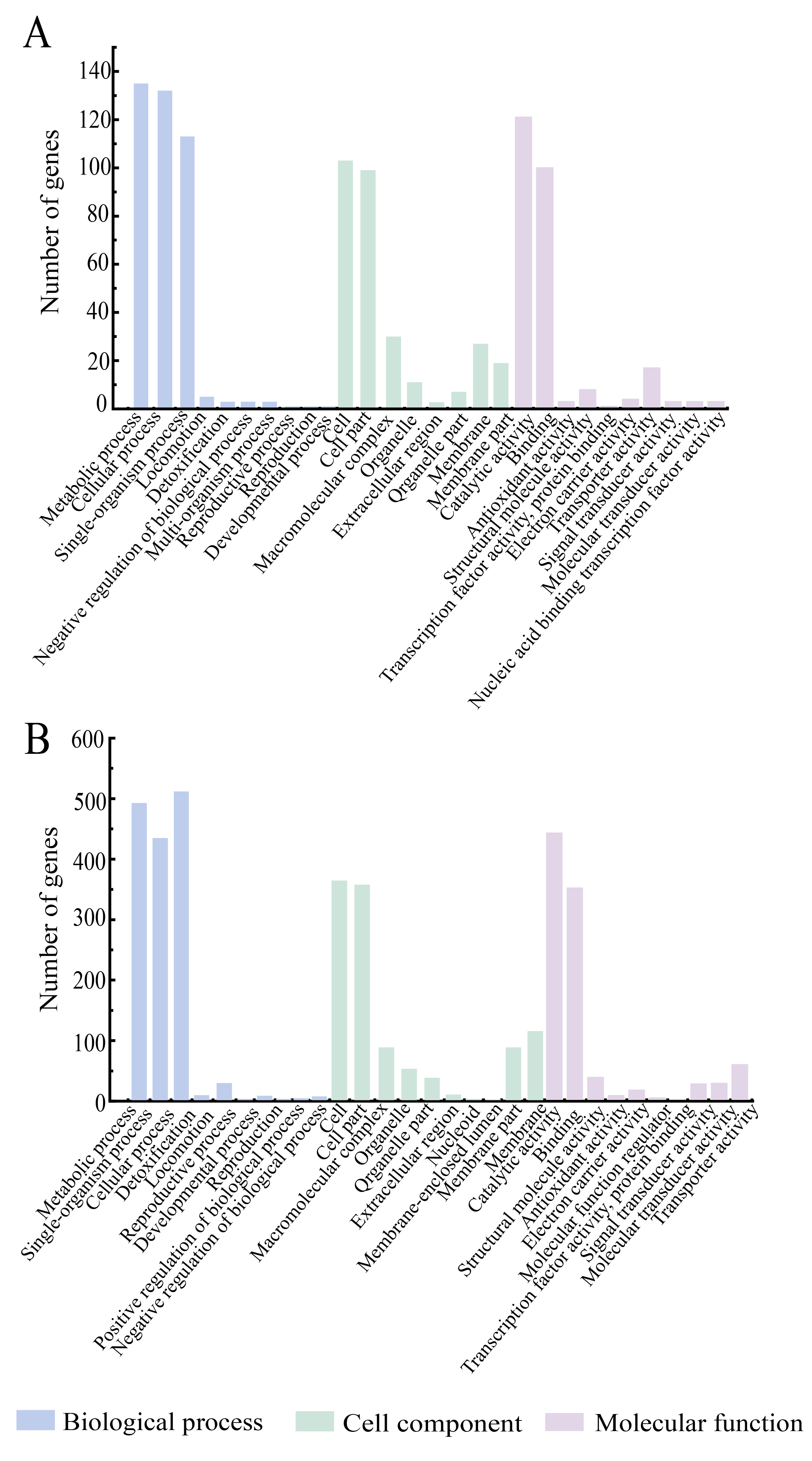

Supplement: Supplementary Figure S1 — Gene functional classification of common and differential intracellular proteins produced by the 11 V. cholerae isolates grown in the eight types of aquatic product matrices. (A) Common proteins. (B) Differential proteins. [file Image_1.TIF]

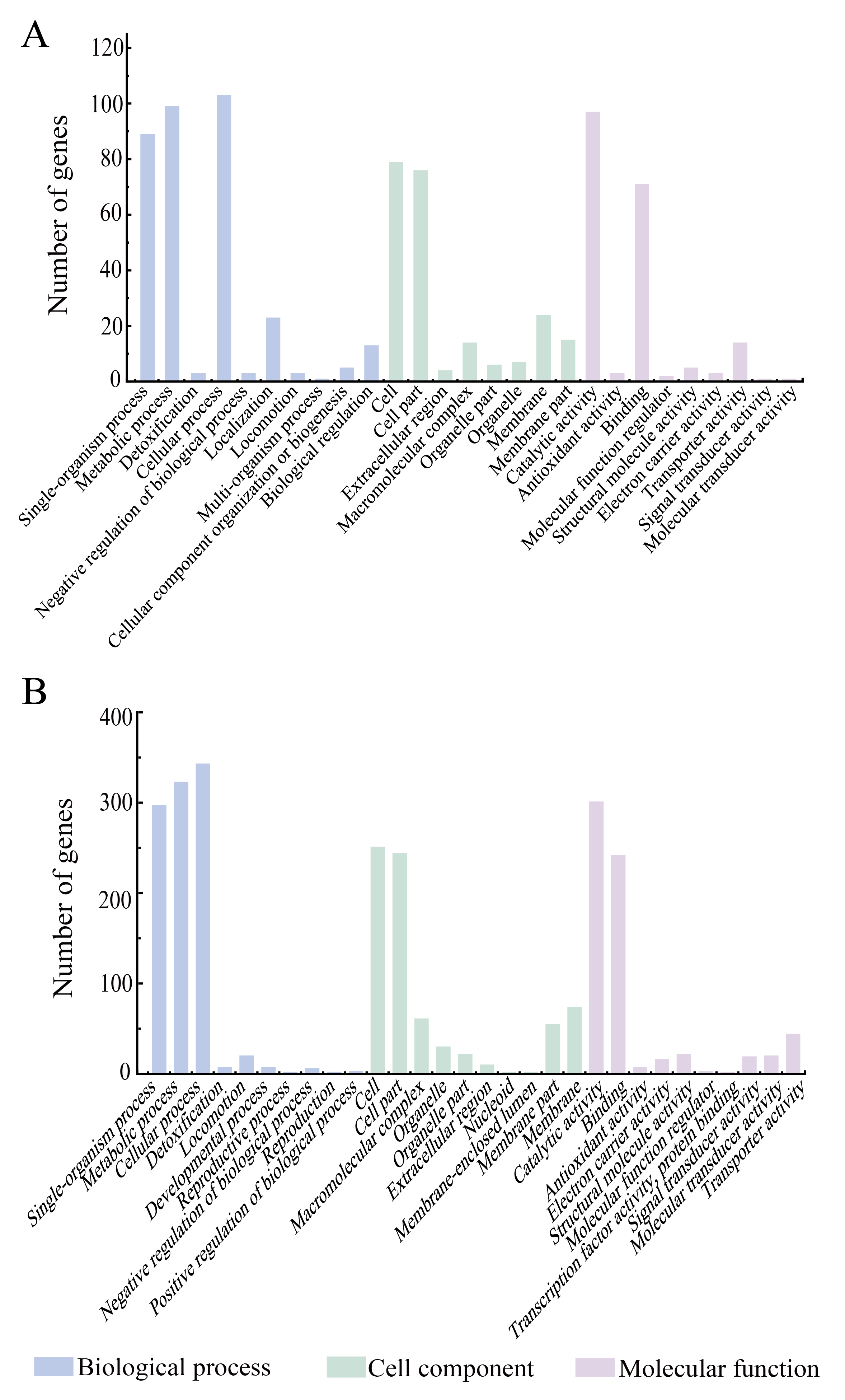

Supplement: Supplementary Figure S2 — Gene functional classification of common and differential intracellular proteins produced by 6 V. cholerae isolates grown in the four types of fish matrices. (A) Common proteins. (B) Differential proteins. [file Image_2.TIF]

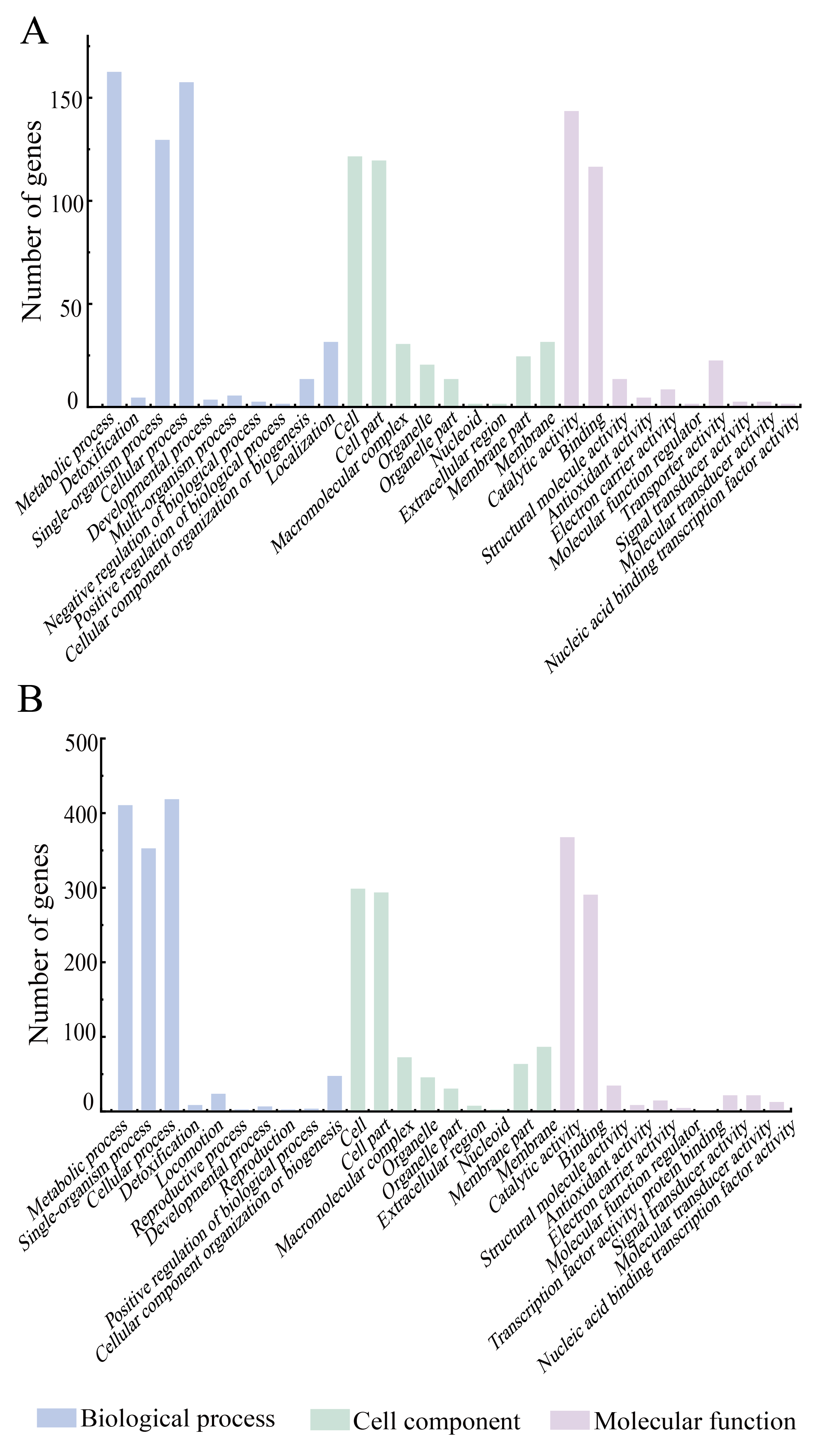

Supplement: Supplementary Figure S3 — Gene functional classification of common and differential intracellular proteins produced by 5 V. cholerae isolates grown in the four types of shellfish matrices. (A) Common proteins. (B) Differential proteins. [file Image_3.TIF]

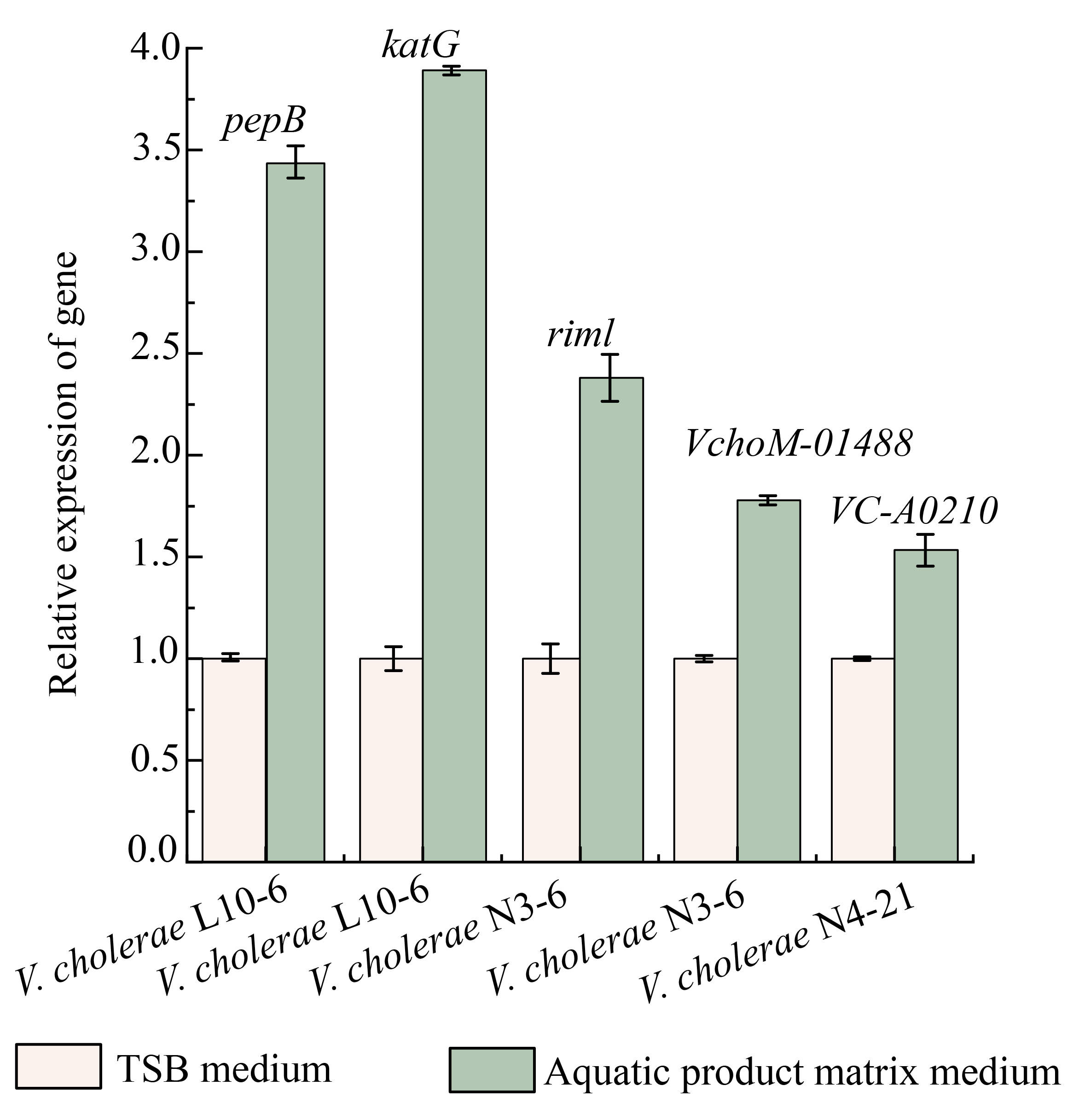

Supplement: Supplementary Figure S4 — The expression of several representative genes by the RT-PCR assay. [file Image_4.TIF]
