# Supplementary material for: Diverse Aquatic Animal Matrices Play a Key Role in Survival and Potential Virulence of Non-O1/O139 Vibrio cholerae Isolates
Source: Front Microbiol. 2022 Jun 21;13:896767. doi: 10.3389/fmicb.2022.896767 (PMC9255913; doi:10.3389/fmicb.2022.896767)
Supplement: Supplementary Table S1 — Genome features of the eleven V. cholerae isolates originating from eight species of aquatic animals. [file Data_Sheet_1.docx]

**Supplementary Materials**

**Table s1** Genome features of the eleven *V. cholerae* isolates originating from eight species of aquatic animals.

| **Feature** | ***V. cholerae* isolate** | | | | | | | | | | |  |
| --- | --- | --- | --- | --- | --- | --- | --- | --- | --- | --- | --- | --- |
|  | b9-50 | B1-31 | B8-16 | J9-62 | L10-6 | N3-6 | N4-21 | N8-56 | N8-88 | Q6-10 | Q10-54 | |
| Genome size (bp) | 4,144,013 | 4,020,504 | 4,014,599 | 4,131,073 | 3,852,021 | 3,924,282 | 3,926,634 | 3,930,222 | 3,923,856 | 3,929,391 | 4,067,586 | |
| G+C content (%) | 47.37 | 47.53 | 47.59 | 47.54 | 47.70 | 47.57 | 47.57 | 47.58 | 47.58 | 47.58 | 47.60 | |
| Total gene | 3,708 | 3,559 | 3,523 | 3,717 | 3,394 | 3,435 | 3,444 | 3,447 | 3,436 | 3,443 | 3,598 | |
| Protein-coding gene | 3,285 | 3,207 | 3,171 | 3,331 | 3,103 | 3,123 | 3,127 | 3,166 | 3,122 | 3,124 | 3,276 | |
| Virulence-related gene | 544 | 535 | 536 | 559 | 536 | 525 | 525 | 525 | 525 | 524 | 544 | |
| Resistance-related gene | 192 | 193 | 191 | 207 | 195 | 186 | 186 | 187 | 186 | 186 | 196 | |
| Secretory protein gene | 344 | 344 | 338 | 343 | 322 | 328 | 328 | 333 | 328 | 328 | 347 | |
| Transporter gene | 872 | 845 | 844 | 887 | 844 | 829 | 828 | 832 | 828 | 831 | 876 | |
| Transmembrane protein gene | 885 | 866 | 872 | 883 | 862 | 829 | 830 | 840 | 829 | 831 | 863 | |
| CRISPR repeat array | 4 | 5 | 5 | 2 | 2 | 3 | 3 | 4 | 2 | 6 | 5 | |
| Prophge gene cluster | 3 | 1 | 2 | 1 | - | 1 | 1 | 1 | 1 | 1 | - | |
| Genes with not hit in COG | 423 | 352 | 352 | 386 | 291 | 312 | 317 | 281 | 314 | 319 | 322 | |

**Table s2** The primers used in the qRT-PCR assay.

| **Primer** | ***V. cholerae* isolate** | **Gene** | **Description of protein function** | **Sequence (5' to 3')** | **Amplicon size (bp)** | **Source** |
| --- | --- | --- | --- | --- | --- | --- |
| F607-F | L10-6 | *katG* | Catalase-peroxidase | GGNCAYTAYGGNGGNYTNATGA | 724 | This study |
| F607-R | L10-6 | *katG* | Catalase-peroxidase | ATNGGCCANARNARNCKNCKNGCYT |  | This study |
| F613-F | L10-6 | *pepB* | Peptidase B | TTYYTNGCNGGNGARGGNTGG | 429 | This study |
| F613-R | L10-6 | *pepB* | Peptidase B | TCNGCNGCNGCNARNGCYTTC |  | This study |
| H2-6-F | N3-6 | *rimI* | [Ribosomal protein S18]-alanine N-acetyltransferase | TGACNACNGTNTTYACNGCNAT | 161 | This study |
| H2-6-R | N3-6 | *rimI* | [Ribosomal protein S18]-alanine N-acetyltransferase | ATNACYTGRTGRCANGCNCC |  | This study |
| S347-F | N3-6 | *VchoM_01488* | Outer membrane protein OmpA | ATGAARAARYTNGCNGCNAT | 321 | This study |
| S347-R | N3-6 | *VchoM_01488* | Outer membrane protein OmpA | ARRCANGCRTCRTCNARCCA |  | This study |
| S629-F | N4-21 | *VC_A0210* | 3'3'-cGAMP-specific phosphodiesterase 2 | CNCAYCAYGARAARTGGGAYGG | 522 | This study |
| S629-R | N4-21 | *VC_A0210* | 3'3'-cGAMP-specific phosphodiesterase 2 | AARTGNCKNCCYTTYTGNGCC |  | This study |
| 16s RNA-F |  |  |  | GACACGGTCCAGACTCCTAC | 179 | Zhu et al., 2020 |
| 16s RNA-R |  |  |  | GGTGCTTCTTCTGTCGCTAAC |  | Zhu et al., 2020 |

**Table S3** Putative extracellular and intracellular virulence-associated proteins secreted and produced by the *V. cholerae* isolates grown in diverse aquatic product matrices.

| **Protein Spot No.** | **Uniprot No.** | **Protein** | **Gene** | **Sequence coverage (%)** | **MW (Da)** | **PI** | **Putative function** | ***V. cholerae* isolate** | **Matrix** | **Reference** |
| --- | --- | --- | --- | --- | --- | --- | --- | --- | --- | --- |
| **Putative extracellular virulence-associated proteins involved in invasion, damage of host cells and tissues, or adhesion** | | | | | | | | | | |
| G2-10 | A0A0H3Q6L9 | icmF-related protein | *VCE_001901* | 0.76 | 134776.32 | 6.79 | -* | b9-50 | *M. antiquata* | Zusman et al., 2004 |
| D2-3,  H2-10,  J2-10,  K2-6 | A0A7X4TA57 | T2SS GspH family protein | *FKR41_09995* | 2.34 | 27737.67 | 7.76 | -* | L10-6,  N3-6,  N8-56,  N8-88 | *A. nobilis,*  *P. undulata,*  *M. quadrangularis Deshayes,*  *M. quadrangularis Deshayes* | Zhang et al., 2018 |
| D2-1 | A0A655SXP5 | Flagellin | *flaD_3* | 5.74 | 12076.18 | 4.12 | -* | L10-6 | *A. nobilis* | Hajam et al., 2017 |
| **Putative intracellular virulence-associated proteins involved in invasion, damage of host cells and tissues, adhesion, chemotaxis, biofilm formation, or regulation of virulence** | | | | | | | | | | |
| S299 | A0A085S6B3 | Glyceraldehyde-3-phosphate dehydrogenase | *gapA* | 67.67 | 35282.65 | 5.80 | Oxidoreductase, NAD, nucleotide-binding | N3-6 | *P. undulata* | Kopeckova et al., 2020 |
| S573 | A0A0K9UUN0 | Glyceraldehyde-3-phosphate dehydrogenase | *VC274080_021129* | 27.33 | 36486.35 | 6.37 | Oxidoreductase, NAD, nucleotide-binding | N4-21 | *P. viridis* | Kopeckova et al., 2020 |
| S742 | A0A0X1KY65 | Glyceraldehyde-3-phosphate dehydrogenase | *VchoM_01232* | 84.29 | 35282.65 | 5.80 | Oxidoreductase | N8-56 | *M. quadrangularis Deshayes* | Kopeckova et al., 2020 |
| S898 | A0A085S6B3 | Glyceraldehyde-3-phosphate dehydrogenase | *gapA* | 81.57 | 35282.65 | 5.80 | Oxidoreductase, NAD, nucleotide-binding | N8-88 | *M. quadrangularis Deshayes* | Kopeckova et al., 2020 |
| F2 | A0A0H3AM92 | Glyceraldehyde-3-phosphate dehydrogenase | *gapA-1* | 75.53 | 35282.65 | 5.80 | Oxidoreductase | B1-31 | *P. pekinensis* | Kosova et al., 2017 |
| F54 | A0A0K9UZF6 | Glyceraldehyde-3-phosphate dehydrogenase | *VC274080_022036* | 71.90 | 35282.65 | 5.80 | Oxidoreductase, NAD, nucleotide-binding | B8-16 | *P. pekinensis* | Kopeckova et al., 2020 |
| F220 | A0A0H3Q291 | Glyceraldehyde-3-phosphate dehydrogenase | *VCE_001537* | 15.67 | 52995.63 | 6.54 | Oxidoreductase | B8-16 | *P. pekinensis* | Kopeckova et al., 2020 |
| F652 | Q9KQJ8 | Glyceraldehyde-3-phosphate dehydrogenase | *VC_2000* | 64.35 | 35282.65 | 5.80 | Oxidoreductase, NAD, nucleotide-binding | Q6-10 | *C. idellus* | Kopeckova et al., 2020 |
| F709 | A0A0X1KY65 | Glyceraldehyde-3-phosphate dehydrogenase | *VchoM_01232* | 75.83 | 35282.65 | 5.80 | Oxidoreductase | Q10-54 | *C. idellus* | Kopeckova et al., 2020 |
| S443 | A0A6N1RT85 | Tol-Pal system protein TolB | *tolB* | 35.78 | 49674.02 | 9.24 | Cell cycle, cell division | N4-21 | *P. viridis* | Godlewska et al., 2009 |
| S850 | A0A5C2AZP4 | Tol-Pal system protein TolB | *tolB* | 21.11 | 49673.04 | 9.14 | Cell cycle, cell division | N8-56 | *M. quadrangularis Deshayes* | Godlewska et al., 2009 |
| F292 | A0A5C2AZP4 | Tol-Pal system protein TolB | *tolB* | 11.11 | 49673.04 | 9.14 | Cell cycle, cell division | B8-16 | *P. pekinensis* | Hirakawa et al., 2019 |
| F691 | A0A0K9UZP5 | Tol-Pal system protein TolB | *tolB* | 13.56 | 49644 | 9.24 | Cell cycle, cell division | Q6-10 | *C. idellus* | Godlewska et al., 2009 |
| s16 | A0A0E4GK44 | Oligopeptide ABC transporter substrate-binding protein OppA | *EYB64_00560* | 26.15 | 61166.35 | 6.14 | -* | b9-50,  N3-6 | *M. antiquata,*  *P. undulata* | Gao et al., 2012 |
| s108 | A0A5C9HJE7 | Oligopeptide ABC transporter substrate-binding protein OppA | *FXF03_16405* | 61.33 | 61182.40 | 6.14 | -* | N4-21,  N8-56,  N8-88 | *P. viridis,*  *M. quadrangularis Deshayes,*  *M. quadrangularis Deshayes* | Gao et al., 2012 |
| s53 | A0A2R8EFA3 | UTP-glucose-1-phosphate uridylyltransferase | *galU* | 21.31 | 32450.31 | 5.79 | Nucleotidyltransferase, transferase | b9-50,  N8-88 | *M. antiquata,*  *M. quadrangularis Deshayes* | Zhou et al., 2020 |
| s55 | A0A0H3Q412 | Endoribonuclease L-PSP | *VCE_001045* | 41.86 | 13657.52 | 5.22 | -* | b9-50,  N4-21 | *M. antiquata,*  *P. viridis* | Reuter et al., 2015 |
|  |  |  |  |  |  |  |  |  |  |  |
| s191 | D7HBC4 | Maltoporin | *VCRC385_02643* | 17.34 | 43248.99 | 4.52 | Porin, ion transport, transport | N4-21,  N8-56 | *P. viridis,*  *M. quadrangularis Deshayes* | Zhu et al., 2020 |
| s217 | A0A3M8JWX0 | HlyD family secretion protein | *F0H40_13260* | 21.01 | 41714.82 | 8.74 | -* | N8-56,  N8-88 | *M. quadrangularis Deshayes*,  *M. quadrangularis Deshayes* | Trent et al., 1998 |
| S58 | A5F0I2 | GroEL protein 1 | *groEL1* | 28.30 | 56198.62 | 5.24 | Chaperone, ATP-binding, nucleotide-binding | b9-50 | *M. antiquata* | O'Shea et al., 2004 |
| S152 | A0A085TAJ7 | Chaperone SurA | *surA* | 17.40 | 48429.43 | 5.51 | Chaperone, isomerase, rotamase chaperone, isomerase, rotamase | b9-50 | *M. antiquata* | Behrens-Kneip, 2010 |
| S431 | A0A0H3QA92 | Chaperone SurA | *surA* | 42.00 | 48487.47 | 5.51 | Chaperone, isomerase, rotamase | N4-21 | *P. viridis* | Behrens-Kneip, 2010 |
| S782 | C3LRH6 | Chaperone SurA | *surA* | 40.84 | 48487.47 | 5.51 | Chaperone, isomerase, rotamase | N8-56 | *M. quadrangularis Deshayes* | Behrens-Kneip, 2010 |
| S917 | A0A0H6PV16 | Chaperone SurA | *surA* | 45.48 | 48457.44 | 5.51 | Chaperone, isomerase, rotamase | N8-88 | *M. quadrangularis Deshayes* | Behrens-Kneip, 2010 |
| S156 | A0A5C9HM23 | Ribonuclease E | *rne* | 9.70 | 117639.53 | 7.02 | Endonuclease, hydrolase, nuclease, tRNA-binding, rRNA processing, tRNA processing, magnesium, zinc metal-binding | b9-50 | *M. antiquata* | Mardle et al., 2019 |
| S554 | A0A7Z8DVI1 | Ribonuclease E | *EYB64_02705* | 10.46 | 117580.51 | 7.02 | Hydrolase | N4-21 | *P. viridis* | Mardle et al., 2019 |
| S173 | A0A0X1L333 | Autoinducer-2 production protein LuxS | *luxS* | 48.26 | 19080.71 | 5.30 | Lyase, autoinducer synthesis, quorum sensing, iron, metal-binding | b9-50 | *M. antiquata* | Teren et al., 2018 |
| S219 | A0A5C2AZF8 | Chemotaxis protein CheW | *F0315_09485* | 39.63 | 18501.73 | 4.48 | Chemotaxis, signal transduction | b9-50 | *M. antiquata* | Huang et al., 2019 |
| S221 | A0A0X1KYB8 | Chemotaxis protein CheY | *VchoM_01298* | 61.54 | 14748.11 | 5.45 | Phosphorelay signal transduction system | b9-50 | *M. antiquata* | Korolik, 2019 |
| S250 | A0A0H3Q0Q0 | Outer membrane protein A | *VCE_003505* | 16.82 | 34285.06 | 5.07 | Porin, ion transport, transport | b9-50 | *M. antiquata* | Nie et al., 2020 |
| S347 | A0A0X1KYV2 | Outer membrane protein OmpA | *VchoM_01488* | 18.69 | 34285.06 | 5.07 | Porin, ion transport, transport | N3-6 | *P. undulata* | Uppalapati et al., 2020 |
| S280 | A0A0X1L4Q0 | DNA-binding protein HU | *VchoM_03553* | 32.22 | 9490.86 | 9.57 | DNA-binding | b9-50 | *M. antiquata* | Stojkova et al., 2019 |
| S368 | A0A7X4PEP8 | Bifunctional proline dehydrogenase/L-glutamate gamma-semialdehyde dehydrogenase PutA | *putA* | 30.13 | 114841.11 | 6.25 | Oxidoreductase | N4-21 | *P. viridis* | Tanner, 2019 |
| S388 | A0A5C2AX76 | ABC transporter substrate-binding protein | *F0315_04640* | 53.67 | 57654.91 | 5.45 | Transmembrane transport | N4-21 | *P. viridis* | Cassio Barreto de Oliveira and Balan, 2020 |
| S490 | A0A1X1LFL9 | ABC transporter substrate-binding protein | *FKR41_00850 FXE88_12515* | 39.24 | 37677.27 | 6.25 | -* | N4-21 | *P. viridis* | Cassio Barreto de Oliveira and Balan, 2020 |
| S549 | A0A085Q5T2 | Amino acid ABC transporter substrate-binding protein | *argT* | 35.27 | 28465.22 | 5.49 | Amino-acid transport, transport | N4-21 | *P. viridis* | Cassio Barreto de Oliveira and Balan, 2020 |
| S400 | Q9K2Y1 | Outer membrane protein TolC | *tolC* | 55.48 | 47750.55 | 5.10 | Antibiotic resistance, transport, virulence | N4-21 | *P. viridis* | Greene et al., 2018 |
| S401 | A0A655XQL6 | Outer membrane protein assembly factor BamA | *yaeT* | 29.19 | 88374.31 | 4.94 | Part of the outer membrane protein assembly complex. Gram-negative-bacterium-type cell outer membrane assembly protein insertion into membrane. | N4-21 | *P. viridis* | Albenne and Ieva, 2017 |
| S770 | A0A0K9UQZ1 | Outer membrane protein assembly factor BamA | *bamA* | 21.54 | 89929.25 | 4.97 | Part of the outer membrane protein assembly complex. Gram-negative-bacterium-type cell outer membrane assembly protein insertion into membrane. | N8-56 | *M. quadrangularis Deshayes* | Albenne and Ieva, 2017 |
| S924 | A0A6B3LBT0 | Outer membrane protein assembly factor BamA | *bamA* | 20.05 | 89872.20 | 4.97 | Part of the outer membrane protein assembly complex. Gram-negative-bacterium-type cell outer membrane assembly protein insertion into membrane. | N8-88 | *M. quadrangularis Deshayes* | Albenne and Ieva, 2017 |
| S426 | A0A6N1RRC6 | VWA domain-containing protein | *HPY05_06445* | 8.04 | 212384.47 | 4.15 | Calcium | N4-21 | *P. viridis* | Springer, 2006 |
|  |  |  |  |  |  |  |  |  |  |  |
| S468 | D7HHD8 | Outer membrane protein assembly factor BamC | *bamC* | 35.10 | 38361.21 | 5.76 | Part of the outer membrane protein assembly complex. Gram-negative-bacterium-type cell outer membrane assembly protein insertion into membrane. | N4-21 | *P. viridis* | Rigel et al., 2012 |
| S972 | A0A6B3LBW2 | Outer membrane protein assembly factor BamC | *bamC* | 25.07 | 38342.16 | 5.68 | Part of the outer membrane protein assembly complex. Gram-negative-bacterium-type cell outer membrane assembly protein insertion into membrane. | N8-88 | *M. quadrangularis Deshayes* | Rigel et al., 2012 |
| S563 | A0A395U9Z5 | X-Pro aminopeptidase | *BC353_16820* | 16.08 | 66600.52 | 5.82 | Aminopeptidase, hydrolase, protease | N4-21 | *P. viridis* | Friedman et al., 2017 |
| S579 | B2CKQ5 | Hemagglutinin/protease regulatory protein | *hapR* | 36.95 | 23644.05 | 5.97 | DNA-binding, hydrolase, protease | N4-21 | *P. viridis* | Wang et al., 2011 |
|  |  |  |  |  |  |  |  |  |  |  |
| S591 | A0A5C9HKW5 | Protease 4 | *sppA* | 9.74 | 67098.67 | 5.00 | Hydrolase, protease | N4-21 | *P. viridis* | Peng et al., 2020 |
| S874 | A0A0H3AH00 | Protease 4 (Endopeptidase IV) (Protease IV) (Signal peptide peptidase) | *sppA* | 8.93 | 67083.66 | 5.04 | Hydrolase, protease | N8-56 | *M. quadrangularis Deshayes* | Peng et al., 2020 |
| S943 | A0A6B3LKX9 | Protease 4(Endopeptidase IV) (Protease IV) (Signal peptide peptidase) | *sppA* | 16.40 | 67111.75 | 5.10 | Hydrolase, protease | N8-88 | *M. quadrangularis Deshayes* | Peng et al., 2020 |
| S592 | A0A5Q6PCV0 | Type VI secretion system tip protein VgrG | *tssI* | 13.37 | 72520.13 | 5.60 | -* | N4-21 | *P. viridis* | Dutta et al., 2019 |
| S714 | A0A544CIF4 | Type VI secretion system tip protein VgrG | *tssI* | 5.11 | 112988.48 | 5.93 | -* | N4-21 | *P. viridis* | Dutta et al., 2019 |
| S593 | A0A5Q6PH83 | Chemotaxis protein CheA | *F0M16_13820* | 9.33 | 84758.98 | 4.73 | -* | N4-21 | *P. viridis* | Xu et al., 2020 |
| S596 | D7H8F4 | Autoinducer 2-binding periplasmic protein LuxP | *VCRC385_00955* | 25.31 | 34824.10 | 4.73 | Sugar transport, transport, calcium, metal-binding | N4-21 | *P. viridis* | Trappetti et al., 2017 |
| S599 | Q9KU89 | Transcription elongation factor GreA | *greA* | 43.95 | 17451.57 | 4.74 | DNA-binding, transcription, transcription regulation | N4-21 | *P. viridis* | Cui et al., 2018 |
| S606 | C3LRZ4 | Phosphoglycerate kinase | *pgk* | 25.00 | 41564.35 | 4.96 | Kinase, transferase, glycolysis, ATP-binding, nucleotide-binding | N4-21 | *P. viridis* | Zhu et al., 2020 |
| S750 | A0A085TAV8 | Phosphoglycerate kinase | *pgk* | 74.94 | 40978.63 | 4.91 | Kinase, transferase, glycolysis, ATP-binding, nucleotide-binding | N8-56 | *M. quadrangularis Deshayes* | Zhu et al., 2020 |
| S621 | A0A5C2AZI5 | Hcp family type VI secretion system effector (Hcp protein) | *hcpA* | 43.02 | 19045.12 | 5.28 | -* | N4-21 | *P. viridis* | Wang et al., 2018b |
| S865 | A0A0K9V0F0 | Hcp protein | *VC274080_022944* | 43.60 | 19059.15 | 5.28 | -* | N8-56 | *M. quadrangularis Deshayes* | Wang et al., 2018b |
| S629 | Q9KMV8 | 3'3'-cGAMP-specific phosphodiesterase 2 (3'3'-cGAMP PDE 2) | *VC_A0210* | 12.64 | 59712.43 | 5.34 | Hydrolase, manganese, metal-binding | N4-21 | *P. viridis* | Deng et al., 2018 |
| S662 | Q9KQU4 | Leucine-responsive regulatory protein | *VC_1904* | 24.39 | 18789.35 | 7.73 | DNA-binding, transcription, transcription regulation | N4-21 | *P. viridis* | Schachterle and Sundin, 2019 |
| S674 | A0A0K9URX0 | UDP-N-acetylglucosamine 2-epimerase | *VC274080_020989* | 17.47 | 41039.66 | 5.50 | Isomerase | N4-21 | *P. viridis* | Wang et al., 2018a |
| S727 | A0A7U8WSM1 | Spermidine/putrescine ABC transporter | *A53_01550* | 11.14 | 42697.24 | 4.96 | -* | N4-21 | *P. viridis* | Zhang et al., 2014 |
| S846 | A0A7U8WRN3 | Spermidine/putrescine ABC transporter | *A53_01546* | 24.35 | 38996.08 | 5.15 | -* | N8-56 | *M. quadrangularis Deshayes* | Zhang et al., 2014 |
| S813 | A0A2V4NVG0 | Gamma-glutamyltransferase | *ggt* | 20.24 | 63075.73 | 5.72 | Acyltransferase, transferase | N8-56 | *M. quadrangularis Deshayes* | Elhosseiny et al., 2020 |
| S887 | H9L3Y0 | IcmF-like protein (Type VI secretion protein IcmF) | *icmF* | 6.44 | 134776.32 | 6.79 | -* | N8-56 | *M. quadrangularis Deshayes* | de Pace et al., 2011 |
|  |  |  |  |  |  |  |  |  |  |  |
| f37 | A0A7U8WQR6 | Peptidoglycan-associated lipoprotein | *A53_01946* | 30.81 | 18490.48 | 4.94 | Lipoprotein | B1-31,  B8-16 | *P. pekinensis*  *P. pekinensis* | Dyke et al., 2020 |
| F4 | A0A0X1KVQ4 | Oligopeptide ABC transporter | *VchoM_00367* | 27.26 | 61139.29 | 6.02 | -* | B1-31 | *P. pekinensis* | Zhou et al., 2018 |
| F20 | A0A0H3AN26 | Peptidase B | *pepB* | 22.07 | 48704.79 | 5.97 | Aminopeptidase, hydrolase, protease. manganese, metal-binding | B1-31 | *P. pekinensis* | Beyzay et al., 2017 |
| F122 | A0A0F0B5L0 | Peptidase B | *pepB* | 31.00 | 46758.68 | 5.58 | Aminopeptidase, hydrolase, protease, manganese, metal-binding | B8-16 | *P. pekinensis* | Beyzay et al., 2017 |
| F398 | D7HEU6 | Peptidase B | *pepB* | 25.45 | 48688.79 | 5.97 | Aminopeptidase, hydrolase, protease, manganese, metal-binding | J9-62 | *C. auratus* | Beyzay et al., 2017 |
| F613 | A0A544C1Y2 | Peptidase B | *pepB* | 21.68 | 46748.64 | 5.58 | Aminopeptidase, hydrolase, protease, manganese, metal-binding | L10-6 | *A. nobilis* | Beyzay et al., 2017 |
| F802 | A0A0X1KUR7 | Peptidase B | *pepB* | 26.13 | 48716.84 | 5.97 | Aminopeptidase, hydrolase, protease, manganese, metal-binding | Q10-54 | *C. idellus* | Beyzay et al., 2017 |
| F21 | Q9KUY4 | Glucose-6-phosphate isomerase (GPI) | *pgi* | 19.09 | 60689.98 | 5.65 | Isomerase, gluconeogenesis, glycolysis | B1-31 | *P. pekinensis* | Richards and Parveen, 2005 |
| F76 | A0A5C2AW56 | Glucose-6-phosphate isomerase (GPI) | *pgi* | 34.18 | 60692.94 | 5.59 | Isomerase, gluconeogenesis, glycolysis | B8-16 | *P. pekinensis* | Richards and Parveen, 2005 |
| F325 | A0A0X1L5M9 | Glucose-6-phosphate isomerase (GPI) | *pgi* | 48.73 | 60689.98 | 5.65 | Isomerase, gluconeogenesis, glycolysis | J9-62 | *C. auratus* | Richards and Parveen, 2005 |
| F671 | A0A6B3LH20 | Glucose-6-phosphate isomerase (GPI) | *pgi* | 22.73 | 60720.99 | 5.59 | Isomerase, gluconeogenesis, glycolysis | Q6-10 | *C. idellus* | Richards and Parveen, 2005 |
| F743 | A0A395TGM3 | Glucose-6-phosphate isomerase (GPI) | *pgi* | 27.09 | 60749.00 | 5.53 | Isomerase, gluconeogenesis, glycolysis | Q10-54 | *C. idellus* | Richards and Parveen, 2005 |
| F30 | A0A395U669 | Flagellin | *BC353_00560* | 23.40 | 39532.11 | 5.05 | -* | B1-31 | *P. pekinensis* | Hajam et al., 2017 |
| F38 | D7HDX8 | Flagellin | *VCRC385_01468* | 21.37 | 40400.48 | 4.85 | Flagellin is the subunit protein which polymerizes to form the filaments of bacterial flagella. | B1-31 | *P. pekinensis* | Hajam et al., 2017 |
| F185 | A0A395U3W1 | Flagellin | *BC353_00565* | 26.53 | 39861.68 | 4.94 | -* | B8-16 | *P. pekinensis* | Hajam et al., 2017 |
| F379 | D7HHC8 | Flagellin | *VCRC385_01435* | 37.14 | 39933.75 | 4.89 | Flagellin is the subunit protein which polymerizes to form the filaments of bacterial flagella. | J9-62 | *C. auratus* | Hajam et al., 2017 |
| F393 | A0A1X1LH93 | Flagellin | *FXE67_12865* | 37.23 | 39459.06 | 5.05 | Flagellin is the subunit protein which polymerizes to form the filaments of bacterial flagella. | J9-62 | *C. auratus* | Hajam et al., 2017 |
| F775 | A0A395U4X0 | Flagellin | *BC353_00825* | 30.50 | 39988.84 | 4.78 | Flagellin is the subunit protein which polymerizes to form the filaments of bacterial flagella. | Q10-54 | *C. idellus* | Hajam et al., 2017 |
| F31 | A0A0H3PZ60 | Catalase-peroxidase (CP) | *katG* | 11.19 | 80649.92 | 5.79 | Oxidoreductase, peroxidase, hydrogen peroxide, heme, iron, metal-binding | B1-31 | *P. pekinensis* | Li et al., 2017 |
| F57 | A0A6B3LLZ5 | Catalase-peroxidase (CP) | *katG* | 32.32 | 80693.98 | 5.79 | Oxidoreductase, peroxidase, hydrogen peroxide, heme, iron, metal-binding | B8-16 | *P. pekinensis* | Li et al., 2017 |
| F607 | D7H923 | Catalase-peroxidase (CP) | *katG* | 12.71 | 80679.95 | 5.79 | Oxidoreductase, peroxidase, hydrogen peroxide, heme, iron, metal-binding | L10-6 | *A. nobilis* | Li et al., 2017 |
| F654 | Q9KRS6 | Catalase-peroxidase (CP) | *katG* | 32.46 | 80649.92 | 5.79 | Oxidoreductase, peroxidase, hydrogen peroxide, heme, iron, metal-binding | Q6-10 | *C. idellus* | Li et al., 2017 |
| F707 | A0A395TQW6 | Catalase-peroxidase (CP) | *katG* | 32.87 | 80659.94 | 5.79 | Oxidoreductase, peroxidase, hydrogen peroxide, heme, iron, metal-binding | Q10-54 | *C. idellus* | Li et al., 2017 |
| F43 | A0A0X1KY92 | Aldehyde-alcohol dehydrogenase | *adhE* | 5.70 | 96231.28 | 6.08 | Oxidoreductase | B1-31 | *P. pekinensis* | Cho et al., 2021 |
| F70 | C3LNY4 | Aldehyde-alcohol dehydrogenase | *adhE* | 24.16 | 96231.28 | 6.08 | Oxidoreductase | B8-16 | *P. pekinensis* | Cho et al., 2021 |
| F803 | A0A5C2AZ90 | Aldehyde-alcohol dehydrogenase | *adhE* | 11.52 | 96187.27 | 6.15 | Oxidoreductase | Q10-54 | *C. idellus* | Cho et al., 2021 |
| F111 | A0A0K9UZM9 | Trigger factor (TF) | *tig* | 30.48 | 47953.37 | 5.00 | Chaperone, isomerase, rotamase, cell cycle, cell division | B8-16 | *P. pekinensis* | De Geyter et al., 2020 |
| F338 | A0A655YZ72 | Trigger factor (TF) | *tig* | 45.33 | 47269.61 | 4.96 | Chaperone, isomerase, rotamase, cell cycle, cell division | J9-62 | *C. auratus* | De Geyter et al., 2020 |
| F719 | A0A0H5Z682 | Trigger factor (TF) | *tig* | 49.19 | 47953.37 | 5.00 | Chaperone, isomerase, rotamase, cell cycle, cell division | Q10-54 | *C. idellus* | De Geyter et al., 2020 |
| F143 | A0A7U8WSP5 | Flagellin B | *A53_02241* | 47.34 | 39516.11 | 5.05 | -* | B8-16 | *P. pekinensis* | Zheng et al., 2017 |
| F614 | P0C6C4 | Flagellin B | *flaB* | 26.86 | 39516.11 | 5.05 | Flagellin is the subunit protein which polymerizes to form the filaments of bacterial flagella. FlaB is not essential for flagellar synthesis and motility (by similarity). | L10-6 | *A. nobilis* | Zheng et al., 2017 |
| F740 | A5F6C4 | Flagellin B | *flaB* | 55.05 | 39516.11 | 5.05 | Flagellin is the subunit protein which polymerizes to form the filaments of bacterial flagella. FlaB is not essential for flagellar synthesis and motility. | Q10-54 | *C. idellus* | Zheng et al., 2017 |
| F148 | Q9KQS7 | ATP-dependent Clp protease ATP-binding subunit ClpX | *clpX* | 26.76 | 46489.68 | 5.06 | Chaperone, ATP-binding, metal-binding, nucleotide-binding, zinc | B8-16 | *P. pekinensis* | Lo et al., 2020 |
| F200 | A0A0X1L4Y0 | Ferritin | *VchoM_03610* | 33.70 | 20667.10 | 4.82 | Iron storage, iron, metal-binding | B8-16 | *P. pekinensis* | He et al., 2019 |
| F629 | A0A0K9UQ90 | Ferritin | *VC274080_020080* | 29.14 | 19870.21 | 4.87 | Iron storage, iron, metal-binding | L10-6 | *A. nobilis* | He et al., 2019 |
| F689 | A0A7U8WLS3 | Ferritin | *A53_00083* | 46.41 | 20667.10 | 4.82 | -* | Q6-10 | *C. idellus* | He et al., 2019 |
| F825 | D7HGX8 | Ferritin | *VCRC385_00075* | 33.70 | 20666.15 | 4.83 | Iron storage, iron, metal-binding | Q10-54 | *C. idellus* | He et al., 2019 |
| F225 | A0A5C9HJ88 | LPS-assembly protein LptD | *lptD* | 9.28 | 89066.24 | 4.68 | Together with LptE, is involved in the assembly of lipopolysaccharide (LPS) at the surface of the outer membrane. | B8-16 | *P. pekinensis* | Lo Sciuto et al., 2018 |
| F498 | A0A395TX30 | LPS-assembly protein LptD | *lptD* | 8.70 | 89791.93 | 4.68 | Together with LptE, is involved in the assembly of lipopolysaccharide (LPS) at the surface of the outer membrane. | J9-62 | *C. auratus* | Lo Sciuto et al., 2018 |
| F263 | A0A0X1L424 | Cyclic AMP receptor protein | *VchoM_03299* | 26.67 | 23635.19 | 8.40 | Receptor | B8-16 | *P. pekinensis* | Ritzert et al., 2019; Manneh-Roussel et al., 2018 |
| F391 | A0A7U8WP57 | Flagellin FlaC | *A53_02296* | 30.50 | 39904.81 | 4.93 | -* | J9-62 | *C. auratus* | Kim et al., 2018 |
|  |  |  |  |  |  |  |  |  |  |  |
| F456 | A0A0H3QAZ7 | Metalloprotease | *VCE_001995* | 8.61 | 101881.22 | 5.06 | Hydrolase, metalloprotease, protease | J9-62 | *C. auratus* | Turk, 2019 |
| F473 | A0A655YFB1 | Amino acid ABC transporter substrate-binding protein | *ERS013199_01542* | 26.77 | 26320.78 | 5.97 | -* | J9-62 | *C. auratus* | Tian et al., 2018 |
| F527 | A0A0E4CHV2 | BON domain-containing protein (Hemolysin) | *BC353_02660* | 25.93 | 20635.60 | 8.97 | -* | J9-62 | *C. auratus* | Liu et al., 2020 |
| F573 | A0A7Z7YCT9 | Aminopeptidase P family protein | *EYB64_11580* | 7.54 | 66593.44 | 5.82 | Aminopeptidase, hydrolase, protease | J9-62 | *C. auratus* | Puente-Rivera et al., 2017 |
| F815 | A0A5Q6PF64 | Aminopeptidase P family protein | *F0M16_16750* | 17.59 | 66608.53 | 6.02 | Aminopeptidase, hydrolase, protease | Q10-54 | *C. idellus* | Puente-Rivera et al., 2017 |
| F577 | A0A0K9UGI6 | Outer membrane protein OmpU | *VC274080_023981* | 10.85 | 36644.55 | 4.46 | -* | J9-62 | *C. auratus* | Li et al., 2018 |
| F690 | D7HDX3 | Outer membrane protein OmpU | *VCRC385_03325* | 23.62 | 36913.98 | 4.63 | -* | Q6-10 | *C. idellus* | Li et al., 2017 |
| F628 | A0A0K9UM14 | Oligopeptidase A | *VC274080_020316* | 7.50 | 77035.15 | 5.12 | Hydrolase, metalloprotease, protease, zinc metal-binding | L10-6 | *A. nobilis* | Anu et al., 2018 |
| F787 | A0A0X1L4F9 | Oligopeptidase A | *VchoM_03463* | 15.59 | 77034.17 | 5.12 | Hydrolase, metalloprotease, protease, zinc metal-binding | Q10-54 | *C. idellus* | Anu et al., 2018 |
| F698 | A0A2P0ZHK8 | Neutral metalloproteinase | *hapA* | 12.15 | 65846.44 | 5.34 | Hydrolase, metalloprotease, protease, zinc | Q6-10 | *C. idellus* | Zhu et al., 2020 |
| F721 | A0A0H3Q198 | Type I secretion TolC | *VCE_003712* | 48.86 | 47750.55 | 5.10 | Transport | Q10-54 | *C. idellus* | Kopping et al., 2019 |

**References**

Albenne, C., and Ieva, R. (2017). Job contenders: roles of the β-barrel assembly machinery and the translocation and assembly module in autotransporter secretion. *Mol. Microbiol*. 106, 505-517. <https://doi.org/10.1111/mmi.13832>

Anu, P.V., Madanan, M.G., Nair, A.J., Nair, G.A., Nair, G.P.M., Sudhakaran, P.R., and Satheeshkumar, P.K. (2018). Heterologous expression, purification and characterization of an oligopeptidase A from the pathogen leptospira interrogans. *Mol. Biotechnol*. 60, 302-309. <https://doi.org/10.1007/s12033-018-0073-8>

Behrens-Kneip, S. (2010). The role of SurA factor in outer membrane protein transport and virulence. *Int. J. Med. Microbiol*. 300, 421-428. <https://doi.org/10.1016/j.ijmm.2010.04.012>

Beyzay, F., Zavaran Hosseini, A., and Soudi, S. (2017). Alpha alumina nanoparticle conjugation to cysteine peptidase A and B: an efficient method for autophagy induction. *Avicenna. J. Med. Biotechnol*. 9, 71-81.

Cassio Barreto de Oliveira, M., and Balan, A. (2020). The ATP-Binding Cassette (ABC) transport systems in *Mycobacterium tuberculosis*: structure, function, and possible targets for therapeutics. *Biology (Basel)*. 9. <https://doi.org/http://doi.org/10.3390/biology9120443>

Cho, S., Kim, G., Song, J.J., and Cho, C. (2021). Cryo-EM structure of *Vibrio cholerae* aldehyde-alcohol dehydrogenase spirosomes. *Biochem. Biophys. Res. Commun*. 536, 38-44. <https://doi.org/http://doi.org/10.1016/j.bbrc.2020.12.040>

Cui, G., Wang, J., Qi, X., and Su, J. (2018). Transcription elongation factor GreA plays a key role in cellular invasion and virulence of francisella tularensis subsp. novicida. *Sci. Rep*. 8, 6895. <https://doi.org/10.1038/s41598-018-25271-5>

De Geyter, J., Portaliou, A.G., Srinivasu, B., Krishnamurthy, S., Economou, A., and Karamanou, S. (2020). Trigger factor is a bona fide secretory pathway chaperone that interacts with SecB and the translocase. *EMBO. Rep*. 21, e49054. <https://doi.org/10.15252/embr.201949054>

de Pace, F., Boldrin de Paiva, J., Nakazato, G., Lancellotti, M., Sircili, M.P., Guedes Stehling, E., Dias da Silveira, W., and Sperandio, V. (2011). Characterization of icmF of the Type VI secretion system in an avian pathogenic *Escherichia coli* (APEC) strain. *Microbiology (Reading)*. 157, 2954-2962. <https://doi.org/http://doi.org/10.1099/mic.0.050005-0>

Deng, M.J., Tao, J., E, C., Ye, Z.Y., Jiang, Z., Yu, J., and Su, X.D. (2018). Novel mechanism for cyclic dinucleotide degradation revealed by structural studies of *Vibrio phosphodiesterase* V-cGAP3. *J. Mol. Biol*. 430, 5080-5093. <https://doi.org/http://doi.org/10.1016/j.jmb.2018.10.010>

Dutta, P., Jijumon, A.S., Mazumder, M., Dileep, D., Mukhopadhyay, A.K., Gourinath, S., and Maiti, S. (2019). Presence of actin binding motif in VgrG-1 toxin of *Vibrio cholerae* reveals the molecular mechanism of actin cross-linking. *Int. J. Biol. Macromol*. 133, 775-785. <https://doi.org/10.1016/j.ijbiomac.2019.04.026>

Dyke, J.S., Huertas-Diaz, M.C., Michel, F., Holladay, N.E., Hogan, R.J., He, B., and Lafontaine, E.R. (2020). The peptidoglycan-associated lipoprotein Pal contributes to the virulence of *Burkholderia mallei* and provides protection against lethal aerosol challenge. *Virulence*. 11, 1024-1040. <https://doi.org/10.1080/21505594.2020.1804275>

Elhosseiny, N.M., Elhezawy, N.B., Sayed, R.M., Khattab, M.S., El Far, M.Y., and Attia, A.S. (2020). γ-Glutamyltransferase as a novel virulence factor of *Acinetobacter baumannii* inducing alveolar wall destruction and renal damage in systemic disease. *J. Infect. Dis*. 222, 871-879. <https://doi.org/http://doi.org/10.1093/infdis/jiaa262>

Friedman, S., Linsky, M., Lobel, L., Rabinovich, L., Sigal, N., and Herskovits, A.A. (2017). Metabolic genetic screens reveal multidimensional regulation of virulence gene expression in *Listeria monocytogenes* and an aminopeptidase that is critical for PrfA protein activation. *Infect. Immun*. 85. <https://doi.org/10.1128/iai.00027-17>

Gao, J., Li, X., Feng, Y., Zhang, B., Miao, S., Wang, L., and Wang, N. (2012). Purification and crystallization of the ABC-type transport substrate-binding protein OppA from *Thermoanaerobacter tengcongensis*. *Biochem. Biophys. Res. Commun*. 423, 45-49. <https://doi.org/http://doi.org/10.1016/j.bbrc.2012.05.067>

Godlewska, R., Wiśniewska, K., Pietras, Z., and Jagusztyn-Krynicka, E.K. (2009). Peptidoglycan-associated lipoprotein (Pal) of Gram-negative bacteria: function, structure, role in pathogenesis and potential application in immunoprophylaxis. *FEMS. Microbiol. Lett*. 298, 1-11. <https://doi.org/10.1111/j.1574-6968.2009.01659.x>

Greene, N.P., Kaplan, E., Crow, A., and Koronakis, V. (2018). Antibiotic resistance mediated by the macB ABC transporter family: a structural and functional perspective. *Front. Microbiol*. 9, 950. <https://doi.org/10.3389/fmicb.2018.00950>

Hajam, I.A., Dar, P.A., Shahnawaz, I., Jaume, J.C., and Lee, J.H. (2017). Bacterial flagellin-a potent immunomodulatory agent. *Exp. Mol. Med*. 49, e373. <https://doi.org/http://doi.org/10.1038/emm.2017.172>

He, J., Fan, K., and Yan, X. (2019). Ferritin drug carrier (FDC) for tumor targeting therapy. *J. Control. Release*. 311-312, 288-300. <https://doi.org/http://doi.org/10.1016/j.jconrel.2019.09.002>

Hirakawa, H., Suzue, K., Kurabayashi, K., and Tomita, H. (2019). The Tol-Pal system of uropathogenic *Escherichia coli* is responsible for optimal internalization into and aggregation within bladder epithelial cells, colonization of the urinary tract of mice, and bacterial motility. *Front. Microbiol*. 10, 1827. <https://doi.org/10.3389/fmicb.2019.01827>

Huang, Z., Pan, X., Xu, N., and Guo, M. (2019). Bacterial chemotaxis coupling protein: structure, function and diversity. *Microbiol. Res*. 219, 40-48. <https://doi.org/http://doi.org/10.1016/j.micres.2018.11.001>

Kim, H.Y., Yu, S.M., Jeong, S.C., Yoon, S.S., and Oh, Y.T. (2018). Effects of *flaC* mutation on stringent response-mediated bacterial growth, toxin production, and motility in *Vibrio cholerae*. *J. Microbiol. Biotechnol*. 28, 816-820. <https://doi.org/http://doi.org/10.4014/jmb.1712.12040>

Kopeckova, M., Pavkova, I., and Stulik, J. (2020). Diverse localization and protein binding abilities of glyceraldehyde-3-phosphate dehydrogenase in pathogenic bacteria: the key to its multifunctionality? *Front. Cell. Infect. Microbiol*. 10, 89. <https://doi.org/10.3389/fcimb.2020.00089>

Kopping, E.J., Doyle, C.R., Sampath, V., and Thanassi, D.G. (2019). Contributions of TolC orthologs to *Francisella tularensis* Schu S4 multidrug resistance, modulation of host cell responses, and virulence. *Infect. Immun*. 87. <https://doi.org/http://doi.org/10.1128/iai.00823-18>

Korolik, V. (2019). The role of chemotaxis during *Campylobacter jejuni* colonisation and pathogenesis. *Curr. Opin. Microbiol*. 47, 32-37. <https://doi.org/http://doi.org/10.1016/j.mib.2018.11.001>

Li, G., Fan, A., Peng, G., Keyhani, N.O., Xin, J., Cao, Y., and Xia, Y. (2017). A bifunctional catalase-peroxidase, MakatG1, contributes to virulence of *Metarhizium acridum* by overcoming oxidative stress on the host insect cuticle. *Environ. Microbiol*. 19, 4365-4378. <https://doi.org/http://doi.org/10.1111/1462-2920.13932>

Li, H., Zhang, W., and Dong, C. (2018). Crystal structure of the outer membrane protein OmpU from *Vibrio cholerae* at 2.2 Å resolution. *Acta. Crystallogr. D. Struct. Biol*. 74, 21-29. <https://doi.org/10.1107/s2059798317017697>

Liu, G., Cheng, C., Guan, X., Ji, Z., Su, J., Zhang, X., and Yu, B. (2020). α-Hemolysin suppresses osteogenesis by inducing lipid rafts accumulation in bone marrow stromal cells. *Food. Chem. Toxicol*. 145, 111689. <https://doi.org/http://doi.org/10.1016/j.fct.2020.111689>

Lo, H.H., Liao, C.T., Li, C.E., Chiang, Y.C., and Hsiao, Y.M. (2020). The clpX gene plays an important role in bacterial attachment, stress tolerance, and virulence in *Xanthomonas campestris* pv. campestris. *Arch. Microbiol*. 202, 597-607. <https://doi.org/10.1007/s00203-019-01772-3>

Lo Sciuto, A., Martorana, A.M., Fernández-Piñar, R., Mancone, C., Polissi, A., and Imperi, F. (2018). *Pseudomonas aeruginosa* LptE is crucial for LptD assembly, cell envelope integrity, antibiotic resistance and virulence. *Virulence*. 9, 1718-1733. <https://doi.org/http://doi.org/10.1080/21505594.2018.1537730>

Mardle, C.E., Shakespeare, T.J., Butt, L.E., Goddard, L.R., Gowers, D.M., Atkins, H.S., Vincent, H.A., and Callaghan, A.J. (2019). A structural and biochemical comparison of ribonuclease E homologues from pathogenic bacteria highlights species-specific properties. *Sci. Rep*. 9, 7952. <https://doi.org/10.1038/s41598-019-44385-y>

Manneh-Roussel, J., Haycocks, J.R.J., Magán, A., Perez-Soto, N., Voelz, K., Camilli, A., Krachler, A.M., and Grainger, D.C. (2018). cAMP receptor protein controls *Vibrio cholerae* gene expression in response to host colonization. *mBio*. 9. <https://doi.org/10.1128/mBio.00966-18>

Nie, D., Hu, Y., Chen, Z., Li, M., Hou, Z., Luo, X., Mao, X., and Xue, X. (2020). Outer membrane protein A (OmpA) as a potential therapeutic target for *Acinetobacter baumannii* infection. *J. Biomed. Sci*. 27, 26. <https://doi.org/http://doi.org/10.1186/s12929-020-0617-7>

O'Shea, Y.A., Reen, F.J., Quirke, A.M., and Boyd, E.F. (2004). Evolutionary genetic analysis of the emergence of epidemic *Vibrio cholerae* isolates on the basis of comparative nucleotide sequence analysis and multilocus virulence gene profiles. *J. Clin. Microbiol*. 42, 4657-4671. <https://doi.org/10.1128/jcm.42.10.4657-4671.2004>

Peng, J., Chen, G., Xu, X., Wang, T., and Liang, H. (2020). Iron facilitates the RetS-Gac-Rsm cascade to inversely regulate protease IV (piv) expression via the sigma factor PvdS in *Pseudomonas aeruginosa*. *Environ. Microbiol*. 22, 5402-5413. <https://doi.org/10.1111/1462-2920.15270>

Puente-Rivera, J., Villalpando, J.L., Villalobos-Osnaya, A., Vázquez-Carrillo, L.I., León-Ávila, G., Ponce-Regalado, M.D., López-Camarillo, C., Elizalde-Contreras, J.M., Ruiz-May, E., Arroyo, R., et al. (2017). The 50kDa metalloproteinase TvMP50 is a zinc-mediated *Trichomonas vaginalis* virulence factor. *Mol. Biochem. Parasitol*. 217, 32-41. <https://doi.org/10.1016/j.molbiopara.2017.09.001>

Reuter, M., Periago, P.M., Mulholland, F., Brown, H.L., and van Vliet, A.H. (2015). A PAS domain-containing regulator controls flagella-flagella interactions in *Campylobacter jejuni*. *Front. Microbiol*. 6, 770. <https://doi.org/10.3389/fmicb.2015.00770>

Richards, G.P., and Parveen, S. (2005). A survey for phosphoglucose isomerase with lysyl aminopeptidase activity in *Vibrionaceae* and non-*Vibrio* pathogens. *Biochim. Biophys. Acta*. 1748, 128-133. <https://doi.org/10.1016/j.bbapap.2004.12.013>

Rigel, N.W., Schwalm, J., Ricci, D.P., and Silhavy, T.J. (2012). BamE modulates the *Escherichia coli* beta-barrel assembly machine component BamA. *J. Bacteriol*. 194, 1002-1008. <https://doi.org/10.1128/jb.06426-11>

Ritzert, J.T., Minasov, G., Embry, R., Schipma, M.J., and Satchell, K.J.F. (2019). The cyclic AMP receptor protein regulates quorum sensing and global gene expression in *Yersinia pestis* during planktonic growth and growth in biofilms. *mBio*. 10. <https://doi.org/http://doi.org/10.1128/mBio.02613-19>

Schachterle, J.K., and Sundin, G.W. (2019). The leucine-responsive regulatory protein Lrp participates in virulence regulation downstream of small RNA ArcZ in *Erwinia amylovora*. *mBio*. 10. <https://doi.org/10.1128/mBio.00757-19>

Springer, T.A. (2006). Complement and the multifaceted functions of VWA and integrin I domains. *Structure*. 14, 1611-1616. <https://doi.org/10.1016/j.str.2006.10.001>

Stojkova, P., Spidlova, P., and Stulik, J. (2019). Nucleoid-associated protein HU: a lilliputian in gene regulation of bacterial virulence. *Front. Cell. Infect. Microbiol*. 9, 159. <https://doi.org/http://doi.org/10.3389/fcimb.2019.00159>

Tanner, J.J. (2019). Structural biology of proline catabolic enzymes. *Antioxid. Redox. Signal*. 30, 650-673. <https://doi.org/10.1089/ars.2017.7374>

Teren, M., Turonova Michova, H., Vondrakova, L., and Demnerova, K. (2018). Molecules autoinducer 2 and cjA and their impact on gene expression in *Campylobacter jejuni*. *J. Mol. Microbiol. Biotechnol*. 28, 207-215. <https://doi.org/http://doi.org/10.1159/000495411>

Tian, M., Bao, Y., Li, P., Hu, H., Ding, C., Wang, S., Li, T., Qi, J., Wang, X., and Yu, S. (2018). The putative amino acid ABC transporter substrate-binding protein AapJ2 is necessary for *Brucella* virulence at the early stage of infection in a mouse model. *Vet. Res*. 49, 32. <https://doi.org/http://doi.org/10.1186/s13567-018-0527-9>

Trappetti, C., McAllister, L.J., Chen, A., Wang, H., Paton, A.W., Oggioni, M.R., McDevitt, C.A., and Paton, J.C. (2017). Autoinducer 2 signaling via the phosphotransferase FruA drives galactose utilization by *Streptococcus pneumoniae*, resulting in hypervirulence. *mBio*. 8. <https://doi.org/10.1128/mBio.02269-16>

Trent, M.S., Worsham, L.M., and Ernst-Fonberg, M.L. (1998). The biochemistry of hemolysin toxin activation: characterization of HlyC, an internal protein acyltransferase. *Biochemistry*. 37, 4644-4652. <https://doi.org/10.1021/bi971588y>

Turk, B.E. (2019). Exceptionally selective substrate targeting by the metalloprotease anthrax lethal factor. *Adv. Exp. Med. Biol*. 1111, 189-203. <https://doi.org/http://doi.org/10.1007/5584_2018_273>

Uppalapati, S.R., Sett, A., and Pathania, R. (2020). The outer membrane proteins OmpA, CarO, and OprD of *Acinetobacter baumannii* confer a two-pronged defense in facilitating its success as a potent human pathogen. *Front. Microbiol*. 11, 589234. <https://doi.org/http://doi.org/10.3389/fmicb.2020.589234>

Wang, G., Kong, J., Cui, D., Zhao, H., Zhao, P., Feng, S., Zhao, Y., and Wang, W. (2018a). Comparative proteomic analysis of two *Ralstonia solanacearum* isolates differing in aggressiveness. *Int. J. Mol. Sci*. 19. <https://doi.org/10.3390/ijms19082444>

Wang, H., Wu, J.H., Ayala, J.C., Benitez, J.A., and Silva, A.J. (2011). Interplay among cyclic diguanylate, HapR, and the general stress response regulator (RpoS) in the regulation of *Vibrio cholerae* hemagglutinin/protease. *J. Bacteriol*. 193, 6529-6538. <https://doi.org/10.1128/jb.05166-11>

Wang, N., Liu, J., Pang, M., Wu, Y., Awan, F., Liles, M.R., Lu, C., and Liu, Y. (2018b). Diverse roles of Hcp family proteins in the environmental fitness and pathogenicity of *Aeromonas hydrophila* Chinese epidemic strain NJ-35. *Appl. Microbiol. Biotechnol*. 102, 7083-7095. <https://doi.org/10.1007/s00253-018-9116-0>

Xu, X., Li, H., Qi, X., Chen, Y., Qin, Y., Zheng, J., and Jiang, X. (2020). cheA, cheB, cheR, cheV, and cheY are involved in regulating the adhesion of *Vibrio harveyi*. *Front. Cell. Infect. Microbiol*. 10, 591751. <https://doi.org/10.3389/fcimb.2020.591751>

Zhang, L., Wen, Y., Li, Y., Wei, X., Yan, X., Wen, X., Wu, R., Huang, X., Huang, Y., Yan, Q., et al. (2014). Comparative proteomic analysis of the membrane proteins of two *Haemophilus parasuis* strains to identify proteins that may help in habitat adaptation and pathogenesis. *Proteome. Sci*. 12, 38. <https://doi.org/10.1186/1477-5956-12-38>

Zhang, Y., Faucher, F., Zhang, W., Wang, S., Neville, N., Poole, K., Zheng, J., and Jia, Z. (2018). Structure-guided disruption of the pseudopilus tip complex inhibits the Type II secretion in *Pseudomonas aeruginosa*. *PLoS. Pathog*. 14, e1007343. <https://doi.org/http://doi.org/10.1371/journal.ppat.1007343>

Zheng, J.H., Nguyen, V.H., Jiang, S.N., Park, S.H., Tan, W., Hong, S.H., Shin, M.G., Chung, I.J., Hong, Y., Bom, H.S., et al. (2017). Two-step enhanced cancer immunotherapy with engineered *Salmonella typhimurium* secreting heterologous flagellin. *Sci. Transl. Med*. 9. <https://doi.org/10.1126/scitranslmed.aak9537>

Zhou, M., Huang, Y., Zhang, Y., Wang, Q., Ma, Y., and Shao, S. (2022). Roles of virulence regulator ToxR in viable but non-culturable formation by controlling reactive oxygen species resistance in pathogen *Vibrio alginolyticus*. *Microbiol. Res*. 254, 126900. <https://doi.org/10.1016/j.micres.2021.126900>

Zhou, B., Yang, Y., Chen, T., Lou, Y., and Yang, X.F. (2018). The oligopeptide ABC transporter OppA4 negatively regulates the virulence factor OspC production of the Lyme disease pathogen. *Ticks. Tick. Borne. Dis*. 9, 1343-1349. <https://doi.org/10.1016/j.ttbdis.2018.06.006>

Zhou, Y., Bu, Z., Qian, J., Chen, Y., Qiao, L., Yang, S., Chen, S., Wang, X., Ren, L., and Yang, Y. (2020). The UTP-glucose-1-phosphate uridylyltransferase of *Brucella* melitensis inhibits the activation of NF-κB via regulating the bacterial type IV secretion system. *Int. J. Biol. Macromol*. 164, 3098-3104. <https://doi.org/10.1016/j.ijbiomac.2020.08.134>

Zhu, Z., Yang, L., Yu, P., Wang, Y., Peng, X., and Chen, L. (2020). Comparative proteomics and secretomics revealed virulence and antibiotic resistance-associated factors in *Vibrio parahaemolyticus* recovered from commonly consumed aquatic products. *Front. Microbiol*. 11, 1453. <https://doi.org/http://doi.org/10.3389/fmicb.2020.01453>

Zusman, T., Feldman, M., Halperin, E., and Segal, G. (2004). Characterization of the *icmH* and *icmF* genes required for *Legionella pneumophila* intracellular growth, genes that are present in many bacteria associated with eukaryotic cells. *Infect. Immun*. 72, 3398-3409. <https://doi.org/http://doi.org/10.1128/iai.72.6.3398-3409.2004>
